# CUX1 Enhances Pancreatic Cancer Formation by Synergizing with KRAS and Inducing MEK/ERK-Dependent Proliferation

**DOI:** 10.3390/cancers13102462

**Published:** 2021-05-18

**Authors:** Heidi Griesmann, Sebastian Mühl, Jan Riedel, Katharina Theuerkorn, Bence Sipos, Irene Esposito, Gregory B. Vanden Heuvel, Patrick Michl

**Affiliations:** 1 Department of Internal Medicine I, Martin Luther University Halle-Wittenberg, D06120 Halle/Saale, Germany; heidi.griesmann@uk-halle.de (H.G.); jan.riedel@uk-halle.de (J.R.); katharina.theuerkorn@uk-halle.de (K.T.); 2 Department of Gastroenterology, Endocrinology and Metabolism, Philipps University Marburg, D35043 Marburg, Germany; Sebastian.Muehl@ukmuenster.de; 3 Institute of Pathology, University of Tuebingen, D72076 Tübingen, Germany; Bence.Sipos@med.uni-tuebingen.de; 4 Institute of Pathology, Heinrich Heine University Duesseldorf, D40225 Düsseldorf, Germany; irene.esposito@med.uni-duesseldorf.de; 5 Department of Biomedical Sciences, Western Michigan University Homer Stryker MD School of Medicine, Kalamazoo, MI 49008, USA; gregory.vandenheuvel@med.wmich.edu

**Keywords:** pancreatic cancer, transgenic mice, KRAS mutation, CUX1, ADAM17, EGFR, MEK-ERK, cell proliferation

## Abstract

**Simple Summary:**

In pancreatic cancer, CUX1 acts as an important mediator of tumor cell proliferation and resistance to apoptosis. Using two different mouse models for the prevalent CUX1 isoforms p200 and p110, we identified p110 CUX1 as the major isoform promoting pancreatic cancer formation in the context of mutant KRAS. We could show an enhanced proliferation by activating and potentiating MEK-ERK signaling via an increased upstream activation of the ADAM17-EGFR axis. This strengthened activation in a KRAS-dependent manner, leading to a dramatically more accelerated formation of invasive PDAC in p110 CUX1 mice within 4 weeks. These results provide the first in vivo evidence for the importance of CUX1 in the development of pancreatic cancer, and highlight CUX1-dependent signaling pathways as potential therapeutic targets.

**Abstract:**

The transcription factor CUX1 has been implicated in either tumor suppression or progression, depending on the cancer entity and the prevalent CUX1 isoform. Previously, we could show that CUX1 acts as an important mediator of tumor cell proliferation and resistance to apoptosis in pancreatic cancer cell lines. However, in vivo evidence for its impact on pancreatic carcinogenesis, isoform-specific effects and downstream signaling cascades are missing. We crossbred two different CUX1 isoform mouse models (p200 CUX1 and p110 CUX1) with KC (KrasLSL-G12D/+; Ptf1aCre/+) mice, a genetic model for pancreatic precursor lesions (PanIN). In the context of oncogenic KRASs, both mice KCCux1p200 and KCCux1p110 led to increased PanIN formation and development of invasive pancreatic ductal adenocarcinomata (PDAC). In KCCux1p110 mice, tumor development was dramatically more accelerated, leading to formation of invasive PDAC within 4 weeks. In vitro and in vivo, we could show that CUX1 enhanced proliferation by activating MEK-ERK signaling via an upstream increase of ADAM17 protein, which in turn led to an activation of EGFR. Additionally, CUX1 further enhanced MEK-ERK activation through upregulation of the serine/threonine kinase MOS, phosphorylating MEK in a KRAS-independent manner. We identified p110 CUX1 as major driver of pancreatic cancer formation in the context of mutant KRAS. These results provide the first in vivo evidence for the importance of CUX1 in the development of pancreatic cancer, and highlight the importance of CUX1-dependent signaling pathways as potential therapeutic targets.

## 1. Introduction

Pancreatic ductal adenocarcinoma (PDAC), one of the most lethal malignancies, is characterized by a high resistance to systemic therapies and associated with a poor 5-year survival rate of approximately 8% [1]. Genetically, more than 90% of all PDAC cases harbor an activating KRAS mutation representing the predominant oncogene in pancreatic tumorigenesis. In transgenic mice, pancreas-specific expression of oncogenic KrasG12D results in formation of pancreatic intraepithelial neoplasias (mPanIN). However, invasive PDAC develops only infrequently and with a long latency [2]. This is in accordance with human data indicating that KRAS mutations can be frequently observed in human pancreata with PanIN lesions being present, but without signs of invasive cancers [3]. Therefore, secondary events such as additional loss of tumor-suppressor genes or activation of further tumor-promoting signaling cascades are generally necessary to foster transformation from preinvasive precursor lesions to invasive cancers.

The transcription factor Cut homeobox 1 (CUX1) belongs to an evolutionarily conserved homeodomain protein family involved in the regulation of numerous cellular processes. In different cancer types, CUX1 has been described either as a tumor suppressor or an oncogenic driver [4,5,6]. Previously, we showed that CUX1 is highly expressed in pancreatic cancer tissues [7,8]. CUX1 is expressed in two major isoforms, the full-length protein p200 and the N-terminally truncated isoform p110, which differ in their DNA binding and transcriptional activity [9]. p200 CUX1 contains four DNA binding domains, three Cut repeats (CR1, CR2 and CR3) and one Cut homeodomain (HD). It is expressed throughout the cell cycle and functions exclusively as transcriptional repressor by interacting transiently with DNA [10,11,12,13,14]. The shorter p110 CUX1 isoform that is proteolytically cleaved by nuclear cathepsin L binds stably to DNA and operates as a transcriptional activator or repressor, depending on the cellular context [4,15,16,17,18]. Several studies using knockdown or genetic inactivation strategies revealed that CUX1 is implicated in cell proliferation [19,20,21], cell migration and invasion [7,17], resistance to apoptosis [8], DNA-damage response [22] and developmental regulation [23,24,25,26]. Overexpression experiments revealed specific roles for the CUX1 isoforms p110 and p200. While p110 CUX1 promotes cell cycle progression, proliferation and cell motility, p200 CUX1 seems to act as transcriptional repressor in terminally differentiated cells of several tissue types [8,24,25,27,28]. In vivo, p200 CUX1 expressed in transgenic mice under the control of a cytomegalovirus (CMV) promoter induced multiorgan hyperplasia and organomegaly [21] accompanied by glomerulosclerosis and interstitial fibrosis of the kidney [29], as well as hepatomegaly associated with inflammation [30]. When expressed under the control of the mouse mammary tumor virus long terminal repeat (MMTV-LTR) promoter, p200 CUX1 induced mammary tumors with a long latency. Mammary tumorigenesis in this model was associated with spontaneously occurring activating mutations within the Kras oncogene (G12V or Q61L) [31]. MMTV-p110 CUX1 mice also developed mammary tumors with a similarly long latency. In these tumors, it is yet unknown whether additional spontaneous Kras mutations occurred [32].

Our previous findings indicate that CUX1 is highly expressed in PDAC and mediates tumor cell proliferation, invasiveness and resistance to apoptosis [8]. However, in its impact in genetic mouse models of Kras-driven pancreatic carcinogenesis, the effects of the p200 and p110 CUX1 isoforms and involved downstream signaling cascades remain to be elucidated. By utilizing two different transgenic mouse models expressing p200 CUX1 or p110 CUX1, respectively, and crossbreeding these mouse lines with KC (KrasLSL-G12D/+; Ptf1aCre/+) mice expressing KRAS in a pancreas-specific manner, we identified p110 CUX1 as major driver of mPanIN progression and pancreatic tumor formation in the context of activated Kras. These results provide the first in vivo evidence in a genetic mouse model underlining the importance of CUX1 in the development of pancreatic cancer.

## 2. Materials and Methods

### 2.1. Cell Culture

All cells were cultured in a humidified atmosphere containing 5% CO_2_ at 37 °C. The amphotropic packaging cell line LinX was maintained in high-glucose DMEM (Life Technologies, Darmstadt, Germany) containing 10% fetal calf serum (FCS, Capricorn Scientific, Ebsdorfergrund, Germany) and 100 µg/mL hygromycin B (Carl Roth, Karlsruhe, Germany). The murine PanIN cell line derived from a KrasLSL-G12D/+, Pdx-1-Cre (KC) mouse with pancreatic precursor lesions was a kind gift from A. Neesse, University of Goettingen, Germany. The human pancreatic cancer cell line Panc-1 was obtained from the German Collection of Microorganisms and Cell Cultures (DSMZ, Braunschweig, Germany). PanIN and Panc-1 cells were retrovirally transduced to generate cells stably expressing p110 CUX1. These cells were cultured in high-glucose DMEM containing 10% FCS and G418 (Carl Roth) at a concentration of 800 µg/mL for PanIN cells and 1400 µg/mL for Panc-1 cells. The nontransformed hTERT-immortalized HPNE cells, isolated from a human pancreatic duct, were a kind gift from M. Buchholz, University of Marburg, Germany. These hTERT-HPNE cells were stably transduced with a constitutively activated Kras (G12D) mutant, followed by a stable transduction to generate cells with or without expression of p110 CUX1 (designated hHPNE-Kras-LV and hHPNE-Kras-p110, respectively). These cell lines were maintained in M3:5 growth medium (4 parts high-glucose DMEM to 1 part M3F (INCELL, San Antonio, TX, USA) supplemented with 5% FBS, puromycin (0.75 µg/mL) (InvivoGen, Toulouse, France) G418 (400 μg/mL) and hygromycin B (50 µg/mL)).

### 2.2. Retroviral Plasmids and Infection

Plasmids for the CUX1 isoforms pLXSN-p200 and pLXSN-p110 were a kind gift from A. Nepveu, McGill University, Montreal. The oncogenic KrasG12D was cloned from the pBabe-puro vector, a kind gift from Channing Der (Addgene plasmid # 58902, Watertown, MA, USA), into the pBabe-hygro, a gift from Hartmut Land, Jay Morgenstern and Bob Weinberg (Addgene plasmid # 1765, Watertown, MA, USA) using the BamHI and SalI restriction sites.

To produce retroviruses, LinX packaging cells were transfected with 5 μg of retroviral vectors; then, 48 and 72 h after transfection, the retrovirus-containing supernatant was harvested, filtered and supplemented with 8 μg/mL of polybrene (Sigma-Aldrich, Darmstadt, Germany). Target cells were transduced by spin infection for 1 h (1500 rpm at 37 °C) and selected with puromycin, G418 and/or hygromycin depending on the cell line.

### 2.3. Western Blot

Cell pellets were lysed in RIPA buffer (50 mM Tris-HCl (pH 7.5), 150 mM NaCl, 0.1% SDS, 1% sodium deoxycholate and 1% Triton X-100) supplemented with protease inhibitor cocktail (Complete, Roche Applied Science, Penzberg, Germany). Protein concentrations were determined with Coomassie brilliant blue (Thermo Scientific, Darmstadt, Germany). Protein samples (10–20 µg of total protein per lane) were separated by SDS-PAGE and transferred to Hybond-P polyvinylidene fluoride membranes (Amersham, Little Chalfont, Buckinghamshire, UK). After blocking, immunoblots were probed with primary antibodies against Cux1 (own antibody prepared as described previously) [7], EGFR, pEGFR (Thr1068), ERK, pERK1/2-Thr202/Tyr204 (Cell Signaling, Frankfurt am Main, Germany), Kras, CCND1 (Proteintech, St. Leon-Rot, Germany), CDK4 (Santa Cruz, Heidelberg, Germany), ADAM17 (Thermo Scientific, Darmstadt, Germany), MOS (LSBio, Seattle, WA, USA) and beta-Actin (Sigma-Aldrich, Darmstadt, Germany), followed by incubation with peroxidase-conjugated secondary antibodies obtained from Amersham. Blots were detected by WesternBright Chemiluminescence Substrate (Biozym, Hessisch Oldendorf, Germany). Quantification by densitometry was done by using Image J software (National Institutes of Health, Bethesda, MD, USA). Relative band intensities were expressed as arbitrary units and normalized to corresponding actin density.

### 2.4. Quantitative Real-Time PCR (qPCR)

Total RNA from cells was isolated and reverse-transcribed using a peqGold Total RNA Kit (Peqlab, Erlangen, Germany) and Omniscript RT Kit (Qiagen, Hilden, Germany). The qPCR reactions were performed using the Luna Universal SYBR Green Supermix (NEB) and the following primers (Biomers, Ulm, Germany): 52032-GTCGGAGGAGTCGGACGAG-3′ and 5′-GCCTTTATTTCCTTGTTTTGCAAA-3′ for RPLP0; 5′-GAAACGAGCGTATCAGCAAA-3′ and 5′-CGGATCCGAGACCTGTAGTT-3′ for CUX1-p110; 5′-GGCCATCAAGCAAGTAAACA-3′ and 5′-TGTTCAGTTCAGCCCAGAAA-3′ for murine MOS; and Hs_MOS_1_SG QuantiTect Primer Assay (Qiagen) for human MOS. SYBR green levels were measured with the ABI PRISM 7500 Sequence Detector System (Applied Biosystems, Darmstadt, Germany), and relative quantitation values were analyzed using the comparative Ct method. The ribosomal protein RPLP0 was used as the internal standard.

### 2.5. Flow Cytometry

Cell cycle analysis was performed after fixation of the cells in 70% ice-cold ethanol using FxCycle PI/RNase Staining Solution (Thermo Scientific, Darmstadt, Germany). Flow cytometry was performed on an LSR II (BD Biosciences, Heidelberg, Germany), and data were analyzed using FlowJo software (BD Bioscience).

### 2.6. Colony Formation Assay

For determining the colony formation efficiency, cells were seeded in complete growth media as following: mPanIN at 1 × 103 cells/10 cm dish and Panc-1 at 500 cells/6 cm dish. After 10–14 days, when colonies were formed, plates were fixed with ice-cold methanol for 10 min and stained with 0.5% crystal violet for 10 min at room temperature. Plates were carefully rinsed with distilled water and air-dried at room temperature. Colonies were counted manually from three independent experiments.

### 2.7. Mouse Experiments

LSL-KrasG12D/+; Ptf1a-Cre (KC) mice [2] and CMV-Cux1+/− (Cux1) mouse lines have been previously described [21,30]. CMV-Cux1+/− animals expressing full-length CUX1 under the control of the CMV promoter were bred with LSL-KrasG12D/+ resulting in LSL-KrasG12D/+; CMV-Cux1+/− (KCux1) mice, which were subsequently mated with Ptf1a-Cre animals to generate LSL-KrasG12D/+; Ptf1a-Cre; CMV-Cux1+/− (KCCux1) mice. The conditional p110 CUX1 mouse line was generated by PolyGene AG (Switzerland) via insertion of the provided p110 CUX1-cDNA into the Rosa26-locus. The used targeting vector for homologous recombination contained an FRT flanked IRES-eGFP reporter gene and loxP flanked neo and STOP cassettes, just between the Rosa26 promoter and the unique cDNA insertion site. Founder animals were backcrossed on a C57BL/6N background for 7 generations. Animals were analyzed at two different time points each: KCCux1 mice after 3 and 15 months, and KCCux1p110 mice around 4 weeks and 3 months. Tissues were obtained at the indicated time points and processed for Western blot or histology. For protein analyses, tissue pieces were snap-frozen in liquid nitrogen. Mice were maintained in a climate-controlled specific pathogen-free (SPF) facility. All animal experiments were approved by the local government authorities and performed according to the guidelines of the animal welfare committee.

### 2.8. Histology and Immunohistochemistry

Mouse pancreata were fixed for 24 h in 3.5% formalin solution (Fischar, Saarbrücken, Germany) and processed for paraffin embedding. Morphological analysis was performed on 4 µm-thick, deparaffinized and rehydrated H&E sections. H&E stainings of KCCux1 mice were evaluated blinded by B.S. regarding the underlying genotype. The disease burden of 3-month-old tumor-free animals was calculated by the percentage of diseased areas (ADMs, PanINs) in relation to areas of the whole tissue sections, at 50× magnification using 3 sections each. Tumor incidence of 15-month-old animals was calculated by the percentage of tumor-bearing animals in relation to the total number of animals. CUX1 immunohistochemistry was performed as follows: deparaffinized sections (4 µm) were rehydrated and quenched in 3% hydrogen peroxide/methanol for 20 min, and antigen retrieval was performed in 10 mM citric acid monohydrate (pH 6.0) for 20 min in a steamer. Blocking was performed in PBS with 2% goat serum and 0.05% BSA for 1 h at room temperature, followed by incubation with the primary antibody against CUX1 (ab54583, Abcam; 1:200), diluted in antibody diluent (Dako, Burlington, ON, Canada) overnight at 4 °C. After incubation with secondary biotinylated antibody for 1 h at RT and addition of ABC solution (all from Vector Laboratories, Burlingame, CA, USA), sections were developed with 3,3′-diaminobenzidine tetrahydrochloride (DAB, Carl Roth, Karlsruhe, Germany). After counterstaining with hematoxylin, slides were mounted with RotiMount (Carl Roth) and examined on an Axio Scope A1 microscope using Axio Vision 4 software. Ki-67 IHC staining was performed as described above; however, antigen retrieval was performed in TE-buffer (pH9) and antibody was used at a dilution of 1:600 (Ki-67 (SP6), Thermo Scientific, Darmstadt, Germany). For pEGFR-Thr1068, antigen retrieval was performed in EDTA-buffer (pH8) and antibody was diluted 1:200. All evaluations of IHC stainings were done in a blinded manner. The evaluation of the CUX1 and pEGFR staining was carried out on a light microscope at 200× magnification. The nuclear staining intensity of CUX1 was assessed in PanIN and ADM areas of 3-month-old noncancerous animals, as well as in the tumor area of 15-month-old tumor-bearing animals of the KCCux1 cohort. The pEGFR staining intensity was assessed in 3-month-old animals of the KCCux1p110 cohort. The following intensity score was used: (−) negative; (+) weak; (++) moderate; (+++) strong. Ki-67 in 3-month-old animals of the KCCux1 cohort was evaluated in 10 high-power fields (HPF) of diseased areas at 400× magnification each.

### 2.9. Statistical Analysis

Data are presented as mean ± standard deviations (SD). All statistical analyses were done by unpaired Student’s *t*-test using GraphPad Prism software. PDAC incidence was analyzed using Fisher’s exact test. The overall survival analysis using Kaplan–Meier curves was performed via log rank test. For all tests, *p* values < 0.05 were considered statistically significant.

## 3. Results

### 3.1. CUX1 Accelerates Pancreatic Tumorigenesis in Kras^G12D^ Mice

**Figure 1:**
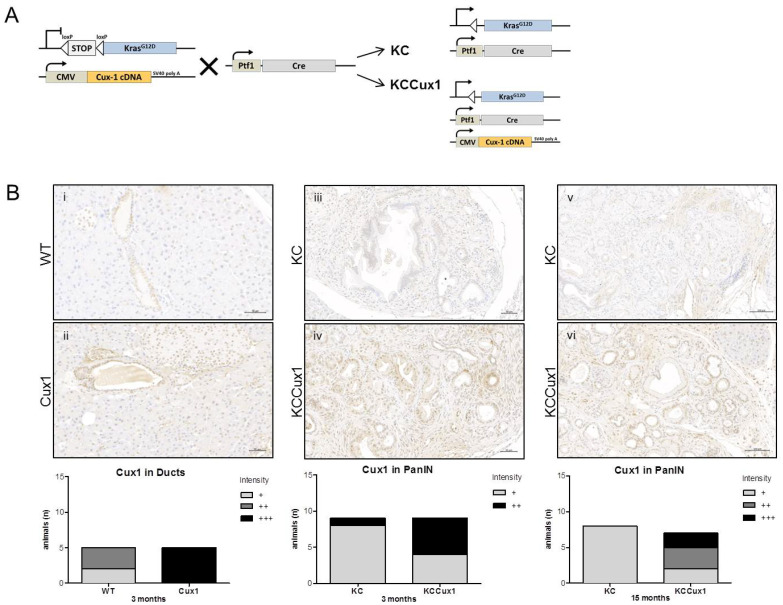
Generation and validation of KCCux1 mice. (**A**) Breeding scheme for the generation of KrasLSL-G12D/+; Ptf1aCre/+; CMV-Cux1+/− mice (p200 CUX1). KrasLSL-G12D/+; CMV-Cux1+/− mice were mated with Ptf1aCre/+ mice to generate Kras-activated KrasLSL-G12D/+; Ptf1aCre/+ and KrasLSL-G12D/+; Ptf1aCre/+; CMV-Cux1+/− animals. (**B**) Representative immunohistochemistry (top) of CUX1 in pancreatic section obtained from 3-month-old WT (**i**), Cux1 (**ii**), KC (**iii**) and KCCux1 (**iv**), as well as 15-month-old KC (**v**) and KCCux1 (**vi**) animals. Original magnification: 200×. Scale bar, 50 µm. Quantification (below) of Cux1 intensity in pancreatic ducts of 3-month-old WT (*n* = 5) and Cux1 (*n* = 5) mice and in mPanIN areas of 3-and 15-month-old KC (*n* = 8) and KCCux1 (*n* = 7) mice. Immunohistochemistry intensity score: + weak, ++ moderate, +++ strong.

To dissect the role of CUX1 in the tumorigenesis and progression of PDAC, we used the KrasLSL-G12D/+; Ptf1aCre/+ (KC) mouse model, which expresses mutant KRAS in a pancreas-specific manner and faithfully recapitulates the formation of preinvasive pancreatic intraepithelial neoplasia (PanIN) lesions [2]. As a transgenic mouse model for p200 CUX1, we used the CMV-Cux1+/− (Cux1) mouse line ubiquitously expressing full-length p200 Cux1 under the control of the CMV promoter. Cux1 mice were mated with KrasLSL-G12D/+ mice and subsequently crossed with Ptf1aCre/+ mice to generateKrasLSL-G12D/+; Ptf1aCre/+ (KC) and KrasLSL-G12D/+; Ptf1aCre/+; CMV-Cux1+/− (KCCux1) animals (Figure 1A).

Immunohistochemistry (IHC) confirmed a strong nuclear signal for CUX1 in pancreatic ducts of 3-month-old Cux1 mice compared to wild-type (WT) mice. Likewise, arising PanIN areas were strongly positive in KCCux1 mice at 3 months of age compared to KC mice. This expression difference persisted in PanIN areas until time of sacrifice at 15 months (Figure 1B). As reported previously by others [21], Western blot of whole pancreas lysates of WT, Cux1, KC and KCCux1 mice did not detect gross differences in CUX1 protein levels, most likely (Figure S1A) due to the exclusively nuclear localization of CUX1, and the fact that whole protein lysates and not specific nuclear extracts were used for protein isolation [21].

**Figure 2:**
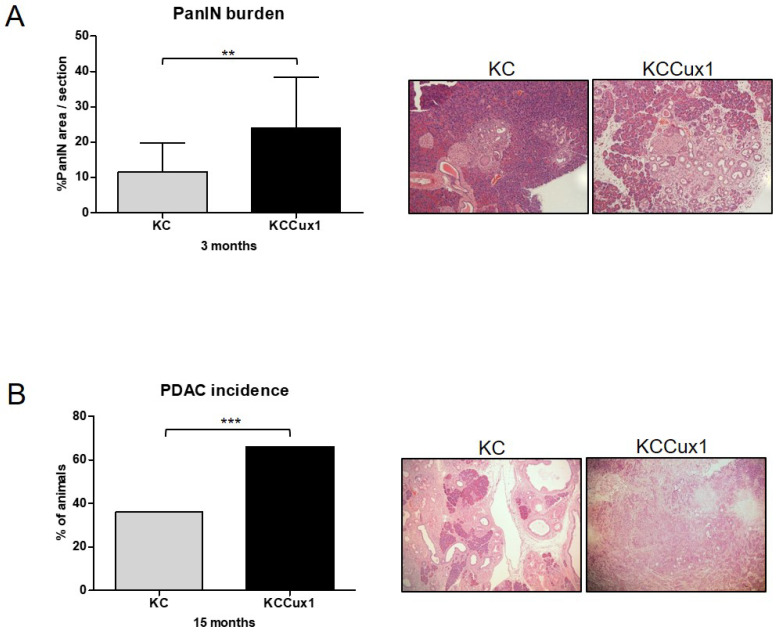
CUX1 accelerates KRAS-driven carcinogenesis and PDAC development. (**A**) Analysis of PanIN burden in 3-month-old KC (grey; *n* = 9) and KCCux1 (black; *n* = 9) mice, with representative H&E stainings (right). ** *p* ≤ 0.0025 by *t*-test. Data are shown as mean ± SD. (**B**) Percentage of 15-month-old tumor-bearing KC and KCCux1 mice, with representative H&E stainings (right). In the KC cohort (grey bar), 9 of 25 mice, and in the KCCux1 cohort (black bar), 20 of 29 mice showed an invasive PDAC. *** *p* ≤ 0.001 by Fisher’s exact test.

Histological analysis of pancreatic tissues revealed a significant increase in PanIN burden after 3 months in KCCux1 versus KC mice (Figure 2A). In addition, a significantly increased number of invasive PDAC could be detected in KCCux1 mice upon sacrifice at month 15 (Figure 2B). Since mice were sacrificed at a defined time point, survival differences were not significant between KCCux1 and KC mice (Figure S2). These data indicated that p200 CUX1 accelerated PanIN formation and development of invasive tumors. However, given the long latency until development of invasive PDAC the impact of p200 CUX1 in this KRAS-driven pancreatic cancer model appeared to be rather modest.

Based on our previous data and reports from others suggesting that the p110 CUX1 variant induces tumor-promoting signaling pathways, we aimed to establish a conditional mouse model expressing p110 CUX1 to investigate its effects on PDAC formation in context with KRAS. For this purpose, we targeted the Rosa26 locus to generate the conditional mouse line Cux1LSL-p110, overexpressing p110 CUX1 after removing the Lox-Stop-Lox (LSL) cassette by Cre-recombination (Figure S3A). Screening of ES-cell clones via Southern blot analysis after digestion with *XbaI* using a Rosa26-specific probe, indicated positive clones showing a signal at 9.8 kbp, which was indicative of the targeted allele, and an additional allele at 4.7 kbp signal, indicative of the WT allele (Figure S3B). All clones showing equal signal intensity for both alleles were selected for generating transgenic mice. To analyze whether p110 CUX1 expression was restricted to the pancreas after pancreas-directed Cre-recombination, we crossed Cux1LSL-p110/+ mice with Ptf1aCre/+ mice.

**Figure 3:**
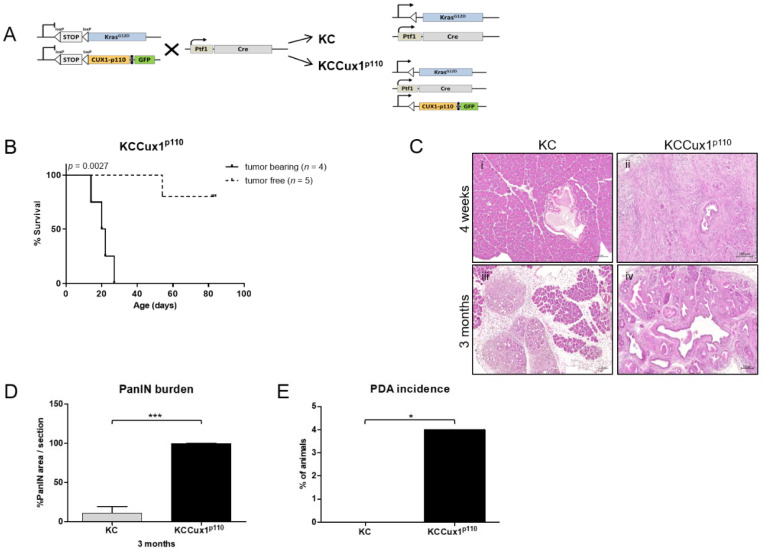
Generation and validation of KCCux1^p110^ mice. (**A**) Breeding scheme for the generation of Kras^LSL-G12D/+^; Ptf1a^Cre/+^; Cux1^LSL-p110/+^ mice. Kras^LSL-G12D/+^; Cux1^LSL-p110/LSL-p110^ mice were mated with Ptf1a^Cre/+^ mice to generate Kras^LSL-G12D/+^; Ptf1a^Cre/+^ and Kras^LSL-G12D/+^; Ptf1a^Cre/+^; Cux1^LSL-p110/+^ animals. (**B**) Survival of tumor-bearing (*n* = 4) and tumor-free (*n* = 5) KCCux1^p110^ mice. Kaplan–Meier curves showed a median survival for tumor-bearing mice of 21 days and for tumor-free mice of 83 days with significant difference by log-rank test, *p* = 0.0027. (**C**) Representative H&E stainings of 4-week-old (left) KC (i) and tumor-bearing KCCux1^p110^ (ii) and 3-month-old (right) KC (iii) and tumor-free mPanIN-bearing KCCux1^p110^ (iv) mice. Scale bar, 100µm. (**D**) Analysis of PanIN burden in 3-month-old KC (grey; *n* = 9) and KCCux1p110 (black; *n* = 5) mice. *** *p* ≤ 0.0001 by *t*-test. Data shown as mean ± SD. (**E**) Percentage of tumor-bearing KC and KCCux1p110 mice. In the KC cohort, 0 of 10 mice, and in the KCCux1p110 cohort (black bar), 4 of 9 mice showed a PDAC. * *p* ≤ 0.02 by Fisher’s exact test.

Western blot analysis clearly confirmed a distinct expression of p110 CUX1 in the pancreas of Ptf1aCre/+; Cux1LSL-110/+ (CCux1p110) mice compared to floxed Cux1LSL-p110/+ (Cux1p110fl) control mice (Figure S3C). This pancreas-specific expression showed no leakiness to liver or lung as main target organs for metastasis in PDAC. Hence, we generated KrasLSL-G12D/+; Cux1LSL-p110/LSL-p110 mice that were subsequently crossed with Ptf1aCre/+ mice to generate KC and KCCux1p110 (KrasLSL-G12D/+; Ptf1aCre/+; Cux1LSL-110/+) animals (Figure 3A). Unexpectedly, the KCCux1p110 mice exhibited an obvious growth retardation soon after birth (Figure S3D), accompanied by a significantly reduced body weight compared to KC, CCux1p110 and Cux1p110fl (Figure S3E). Interestingly, approximately half of the KCCux1p110 mice died within the first four weeks (Figure 3B) and had developed invasive pancreatic cancers (G2) with dedifferentiated G3 areas. Surviving mice were sacrificed at month 3 and showed numerous acinar-to-ductal metaplasia (ADM) formations, as well as a massive burden of grade 1–3 PanIN lesions (Figure 3C). In comparison, age-matched KC mice showed no indications of invasive pancreatic cancer, but—as expected and described previously—also exhibited ADM and grade 1 PanIN formation, but to a lesser extent compared to KCCux1p110 mice (Figure 3D). Therefore, PDAC incidence was significantly enhanced in KCCux1p110 mice compared to KC mice (Figure 3E).

These data indicated that p110 CUX1 conditionally expressed in the pancreas together with oncogenic Kras induced a drastically accelerated pancreatic tumorigenesis in vivo.

### 3.2. CUX1 Enhances Proliferation Pathways during Tumorigenesis

**Figure 4:**
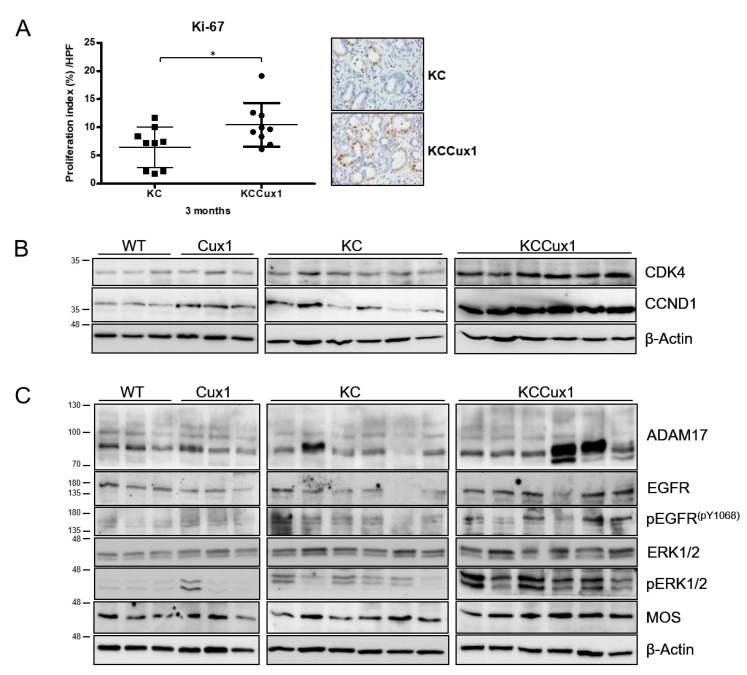
CUX1 potentiates KRAS-driven proliferation during PanIN progression. (**A**) Analysis of Ki-67 in PanIN areas of 3-month-old KC (grey; *n* = 9) and KCCux1 (black; *n* = 9) mice, with representative immunohistochemistry stainings (right). * *p* ≤ 0.03 by *t*-test. Data shown as mean ± SD. Symbols: dots indicate Ki-67-positive cells (%) per high-power field from each animal; the black horizontal line represents the mean percentage for each group. (**B**,**C**) Western blot analysis of whole pancreas protein lysates from 3-month-old WT, Cux1, KC and KCCux1 mice for proliferation markers CCND1 and CDK4 (**B**), as well as upstream signaling effectors EGFR, pEGFR and ADAM17 and their downstream effectors ERK, pERK and MOS (**C**). Actin served as control. The uncropped Western Blot images can be found in Figure S9 and the densitometry analysis can be found in Figure S10.

We and others previously described CUX1 as central transcriptional regulator of tumor cell proliferation. To analyze whether the increased PanIN burden and tumor formation seen in both mouse models was paralleled by an enhanced proliferation index in the pancreatic epithelial compartment, we determined the proliferation marker Ki-67 in pancreatic tissues by immunohistochemistry. In KCCux1 mice, the accelerated malignant transformation was accompanied by a significantly increased proportion of Ki-67-positive cells in the PanIN lesions of 3-month-old KCCux1 mice compared to KC mice (Figure 4A). This increased proliferation was paralleled by enhanced protein levels of cyclin-dependent kinase 4 (CDK4) and cyclin D1 (CCND1) in pancreatic protein lysates from KCCux1 mice as compared to KC, Cux1 and WT mice, consistent with accelerated cell cycle progression (Figure 4B). In pancreatic protein lysates of 3-month-old KCCux1p110 mice, we also could confirm markedly enhanced expression levels of the proliferation markers CDK4 and CCND1 compared to control (Cux1p110fl), CCux1p110 and KC mice (Figure S4A).

Interestingly, we detected a markedly increased phosphorylation of ERK1/2 in KCCux1 mice compared to KC mice, despite constitutively active KRAS being present in both KC and KCCux1 mice (Figure 4C). This suggests that alternate pathways are induced by CUX1 that activate ERK signaling by bypassing of synergizing with active KRAS. Previously, we could show that CUX1-dependent proliferation is induced by EGF-signaling [33]. In addition, Ardito et al. described that formation of preneoplastic lesions and enhanced proliferation via ERK activation in KC mice are accompanied by EGFR upregulation and activation. The latter is facilitated by ADAM17, a sheddase involved in the processing of various signaling molecules, including EGFR ligands at the surface of the cell, leading to an increase in availability and activity of these ligands [34]. To elucidate the CUX1-induced downstream pathways triggering pancreatic cell proliferation, we therefore focused on potential alterations in EGF-dependent signaling cascades.

In pancreatic lysates of both KCCux1 (Figure 4C) and KCCux1p110 (Figure S4B) mice, we were able to detect increased EGFR protein levels compared to KC and control mice, with a concomitant phosphorylation of EGFR (pY1068) indicating its enhanced activation in KCCux1 mice. In KCCux1p110 mice, phosphorylation of EGFR (pY1068) was not detectable by Western blot. However, IHC for phosphorylated EGFR (pY1068) showed a clearly increased signal in KCCux1p110 mice compared to KC and control mice (Figure S5A). Interestingly, we could detect markedly increased protein levels of ADAM17 in both KCCux1 and KCCux1p110 mice compared to KC and control mice, representing a potential mechanism for CUX1-induced EGFR activation (Figure 4C and Figure S4B). In KCCux1p110 mice, we could provide evidence that ADAM17 upregulation is associated with increased levels of the EGFR ligand amphiregulin (AREG), consistent with the notion that CUX1-induced ADAM17 leads to enhanced levels of EGFR ligands such as AREG (Figure S4B). Enhanced EGFR activation by its ligands has been described to trigger MEK-ERK activation irrespectively of the presence of mutant KRAS, resulting in enhanced cell proliferation [34,35].

We also investigated if CUX1 was able to induce MEK-ERK phosphorylation via effectors operating downstream of KRAS. Besides RAF kinases as well-known activators of MEK, the serine/threonine kinase MOS has been described as potent inducer of persistent MEK-ERK activation in MOS-transformed NIH3T3 cells [36]. Indeed, we detected a marked upregulation of MOS protein levels in KCCux1 mice compared to KC and control mice (Figure 4C). In KCCux1p110 mice, MOS upregulation on the protein level was not as unambiguous (Figure S4B). Therefore, we confirmed a significantly increased MOS mRNA in KCCux1p110 mice compared to control mice and an apparent trend compared to KC mice (Figure S5C). These findings in murine pancreatic tissues indicated that CUX1 in the context of mutant KRAS accelerated proliferation and tumor development via MEK-ERK-dependent induction of proliferation pathways. MEK-ERK activation is caused by at least two mechanisms: first, an ADAM17-dependent activation of upstream EGFR signaling; and second, upregulation of the kinase MOS that is able to activate MEK-ERK in a KRAS-independent manner.

### 3.3. p110 CUX1 Is Crucial for Enhancing KRAS-Driven Tumorigenesis

CUX1 is expressed in two relevant variants: Full-length p200 CUX1 (p200) being able to transiently bind to DNA and p110 CUX1 (p110) that is involved in both transcriptional activation and repression of several genes during S-phase via stable DNA binding [15]. p110 CUX1 is generated by proteolytical processing of p200 CUX1 via the endopeptidase cathepsin L, as described before in several tumor models [32,37,38,39]. Based on these data, we aimed to examine which CUX1 variant was predominantly responsible for the observed tumor-promoting phenotype in KCCux1 mice.

**Figure 5:**
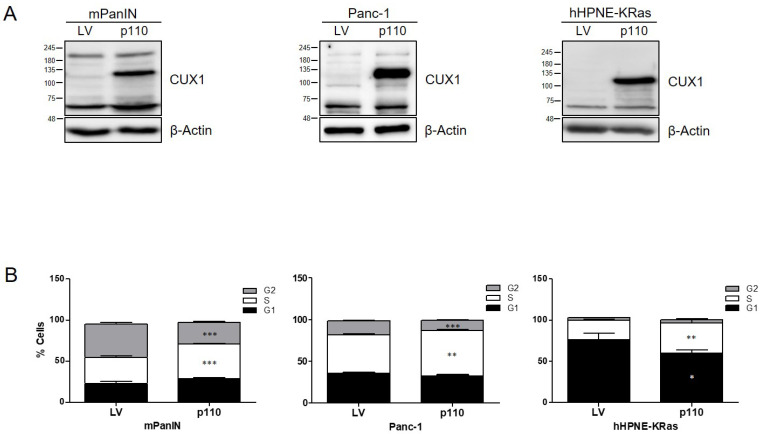
p110 CUX1 enhances cell cycle progression in KRAS-mutant pancreatic cell lines. (**A**) Analysis of p110 CUX1 protein levels in whole cell lysates of stably transduced mPanIN, Panc-1 and hHPNE-KRAS cells. Actin served as control. (**B**) Cell cycle analysis by flow cytometry showing increased S-phase progression in p110 CUX1 overexpressing mPanIN and hHPNE-KRAS cells compared to empty vector cells (LV). Graphs represent the mean percentage ± SD of cells in G1 (black), S (white) and G2 (grey) phase and are representative of three independent experiments. *** *p* ≤ 0.0009; ** *p* ≤ 0.0014–0.009; * *p* ≤ 0.03. The uncropped Western Blot images can be found in Figure S9 and the densitometry analysis can be found in Figure S10.

After transducing the human Panc-1 tumor cell line with full-length p200 CUX1, we could verify by Western blot analysis that p200 CUX1 is—at least in part—processed into the p110 variant (Figure S6A), most likely via proteolytic cleavage by cathepsin L, as reported before in breast and prostate cancer [32,37,38,39]. To test the hypothesis that p110 was synergizing with KRAS to promote pancreatic tumor formation, we generated stable p110 CUX1-expressing cell lines using murine mPanIN cells derived from KC mice, human Panc-1 cells and primary human duct-derived hTERT-HPNE cells as an in vitro model for preneoplastic PanINs. HPNE cells were additionally transduced with KRASG12D generating hTERT-HPNE-KRASG12D cells (hHPNE-KRAS) to investigate the role of p110 in the context of oncogenic KRAS. We could confirm strong expression levels of p110 CUX1 protein in all three cell lines (Figure 5A), which were also present on the mRNA level (Figure S7A). While no morphological differences were observed between empty vector and p110 CUX1-expressing cells, cell cycle analysis revealed a significantly enhanced S-phase progression in p110 CUX1-expressing cell lines compared to their respective control cells (Figure 5B).

**Figure 6:**
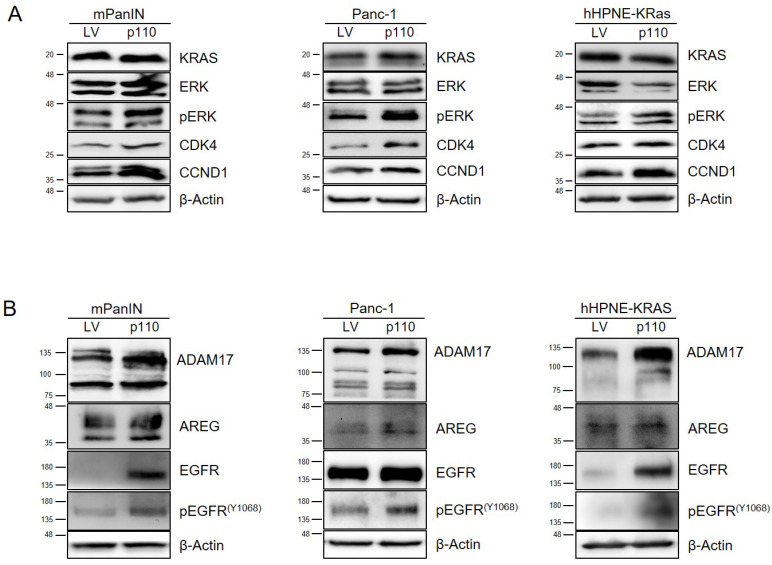
p110 CUX1 potentiates proliferation via upregulation of ADAM17 and increased EGFR activation in KRAS-mutant pancreatic cell lines. (**A**,**B**) Western blot analysis of whole protein cell lysates from stable transduced mPanIn, Panc-1 and hHPNE-KRas cells. Actin served as control. (**A**) Overexpression of p110 potentiated the protein levels of the cell proliferation markers pERK, CDK4 and CCND1 compared to empty vector (LV) control cells. Phosphorylated ERK and total ERK were determined using specific antibodies. (**B**) Compared to empty vector (LV) control cells, overexpression of p110 was associated with increased protein levels of total EGFR and phosphorylated EGFR (Y1068), as well as ADAM17, an EGFR ligand sheddase. Phosphorylated EGFR and total EGFR were determined using specific antibodies. The uncropped Western Blot images can be found in Figure S9 and the densitometry analysis can be found in Figure S10.

To confirm that the CUX1 effector pathways detected in both mouse models in vivo were indeed induced by p110 CUX1 and operative in vitro, we examined expression levels of proliferation-related genes that were upregulated in pancreatic tissues of KCCux1 mice. We confirmed a p110 CUX1-dependent increase in ERK phosphorylation in all three cell lines, accompanied by elevated protein levels of CCND1 and CDK4 and consistent with enhanced G1/S progression (Figure 6A). In line with our in vivo findings, p110 CUX1-overexpressing cells exhibited a significantly increased colony formation capacity, indicating an enhanced ability for anchorage-independent growth mediated by p110 CUX1 (Figure S8).

**Figure 7:**
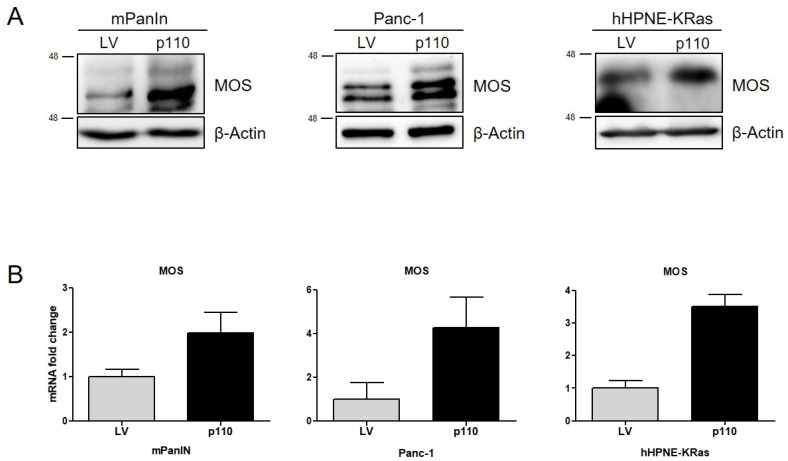
p110 CUX1 increases MOS in KRAS-mutant pancreatic cell lines. (**A**) Western blot analysis of whole protein cell lysates from stable transduced mPanIn, Panc-1 and hHPNE-KRAS cells. Compared to empty vector (LV) control cells, overexpression of p110 CUX1 increased protein levels of MOS in all three pancreatic cell lines. Actin served as control. (**B**) Representative quantitative RT-PCR analysis of MOS mRNA in empty vector (LV) and p110 CUX1 overexpressing mPanIN, Panc-1 and hHPNE-KRAS cells. MOS expression was normalized to the ribosomal protein RPLP0 as internal standard. The uncropped Western Blot images can be found in Figure S9 and the densitometry analysis can be found in Figure S10.

Our data for KCCux1 mice, as well as other studies on PDAC development [40], suggest that oncogenic KRASG12D cooperates with EGFR signaling induced by ADAM17, which leads to enhanced proliferation and formation of preneoplastic lesions. To test this hypothesis in vitro and prove that p110 CUX1 was acting via the ADAM17-EGFR axis, we evaluated p110 CUX1-dependent alterations in protein levels of EGFR, phosphorylated EGFR as well as its ligand sheddase ADAM17 in all three cell lines in vitro. We could confirm our in vivo data by demonstrating that p110 CUX1 markedly increased EGFR levels in preneoplastic mPanIN and hHPNE-KRAS cells exhibiting very low baseline EGFR protein expression (Figure 6B). In contrast, Panc-1 cells representing an established PDAC cell line already showed a strong basal EGFR protein expression, which was only slightly increased by p110 CUX1. However, a CUX1-dependent increase in EGFR phosphorylation (pY1068) was persistently observed in all three cell lines (Figure 6A). As detected in vivo, we could also confirm upregulation of the EGFR ligand sheddase ADAM17 in all three p110-overexpressing cell lines compared to the control cells (Figure 6B), corroborating the hypothesis that ADAM17 contributed to the transactivation of EGFR signaling. In addition, we were also able to verify in vitro that MOS was transcriptionally upregulated in a CUX1-dependent manner: p110 CUX1 overexpression was associated with a marked upregulation of MOS protein (Figure 7A) and mRNA (Figure 7B) compared to control cells.

**Figure 8:**
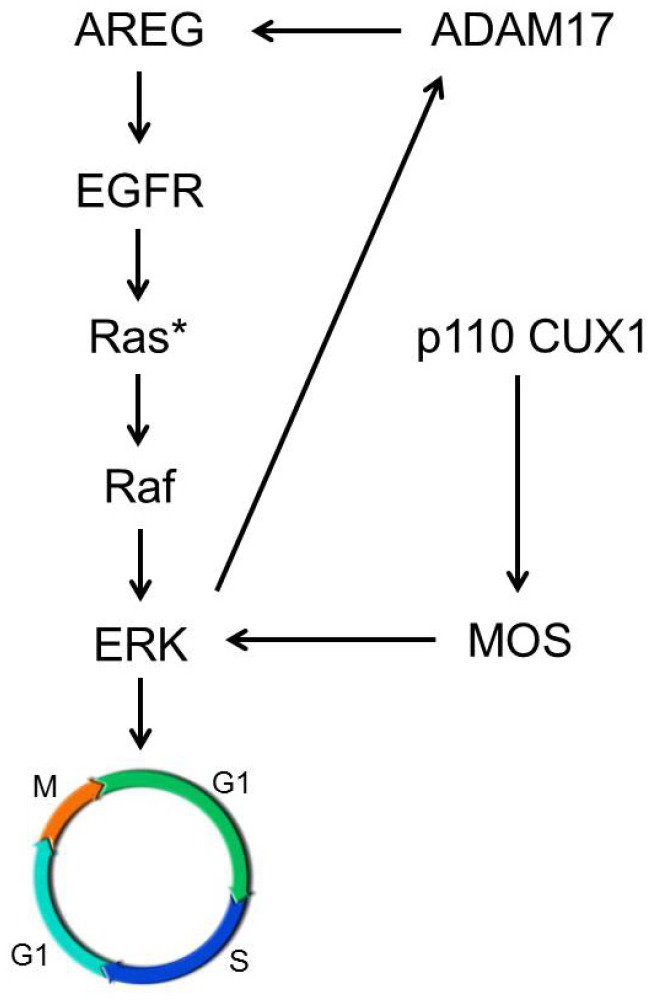
Schematic depiction p110 CUX1 effect on EGFR–MEK-ERK signaling. p110 CUX1 synergizes with EGFR-KRAS^mut^ signaling by reinforcing MEK-ERK signaling via upregulation of the EGF ligand sheddase ADAM17 and the serine/threonine kinase MOS.

These results suggest that in the context of oncogenic KRAS p110 CUX1 increases cell proliferation in vitro by inducing the EGFR axis via upregulation of ADAM17 and by further enhancing MEK-ERK activity via transcriptionally inducing the serine/threonine kinase MOS, supporting our in vivo data in KCCux1 mice (Figure 8).

## 4. Discussion

In this study, we could demonstrate that CUX1 plays an important role in enhancing pancreatic carcinogenesis in vitro and in vivo. The p110 CUX1 isoform increased proliferation in the context of oncogenic KRAS by activating the EGFR axis, at least in part via upregulation of the EGFR ligand sheddase ADAM17. In addition, p110 CUX1 further enhanced MEK-ERK activity via transcriptionally inducing the serine/threonine kinase MOS. In vivo, CUX1 accelerated pancreatic tumorigenesis by accelerating KRAS-driven development of pancreatic precursor lesions and enhancing development of invasive cancers in two different genetic mouse models in which either full-length CUX1 was expressed under the control of a non-cell type-restricted promoter (CMV) or the p110 CUX1 variant conditionally expressed under the control of a pancreas-specific promoter (Ptf1a). In the conditional p110 CUX1 mouse model, tumor development was much more accelerated compared to the model with ubiquitous expression of p200 CUX1. To our knowledge, this is the first report describing protumoral effects of CUX1 in genetic mouse models of pancreatic cancer.

Our data are in line with several reports in the literature describing CUX1 as important trigger of carcinogenesis in various solid tumor entities including glioma [41], neuroblastoma [42] and breast cancer [38]. In contrast to solid tumors and for reasons not entirely understood, CUX1 may act as tumor suppressor in myeloid malignancies in which it is frequently inactivated [43].

Our findings indicating a synergistic cooperativity between CUX1 and mutant KRAS are in concordance with several recent reports from the literature: Ramdzan et al. reported that RAS-transformed tumor cells require CUX1-dependent repair of oxidative DNA damage [31]. Using mammary and lung tumor models, the authors could further show that elevated expression of CUX1 prevents Ras-induced senescence. In addition, CUX1 knockdown was lethal in human cancer cells expressing oncogenic RAS [31]. In analogy to these findings, we had previously observed that pancreatic cancer cells harboring oncogenic KRAS mutations undergo apoptotic cell death upon CUX1 knock-down [8].

Interestingly, Wang et al. demonstrated that mutant KRAS promotes cathepsin L expression and activity thereby enhancing lung cancer cell invasion through Cathepsin L/CUX1-mediated EMT pathways [39]. Our data and reports from literature [44] support these findings indicating that KRAS-mutant pancreatic cancer cells harbor significant cathepsin L activity. In vitro and in vivo, we could show that the p110 CUX1 variant, resulting from proteolytic cleavage of full-length p200 CUX1 by cathepsin L, was active in PDAC and enhanced tumor development in concert with oncogenic Ras. In our p110 CUX1 mouse model, tumor progression was much more accelerated compared to mice expressing full-length CUX1. Varying CUX1 expression levels and patterns due to the different promoters might contribute to these phenotypic differences. However, the much more aggressive phenotype seen in p110 CUX1 mice supported the notion that this CUX1 cleavage product had a strong tumor-promoting impact. KCCux1p110 mice showed a drastically accelerated tumor development compared to mice with oncogenic Ras alone, with a high lethality within 4 weeks postpartum. Interestingly, KCCux1p110 mice without invasive PDAC were also considerably smaller than their comparative littermates. Based on the significant PanIN burden already present in the postnatal period, we hypothesize that exocrine pancreatic insufficiency contributes to the growth retardation.

Taken together, both genetic in vivo models suggest that CUX1 acts as an important accelerator of tumor development, with p110 CUX1 as cleavage product of oncogenic cathepsin L activity being the most active CUX1 variant.

In line with our findings, the importance of p110 CUX1 as a facilitator of tumor development and progression has recently been demonstrated in several other tumor models, including breast cancer and glioma [17,45,46]. In prostate cancer, the different CUX1 variants even seem to mediate antagonistic effects depending on cathepsin L activity: In androgen-sensitive tumors, cathepsin L is highly expressed and cleaves CUX1 into the tumor-promoting p110 isoform. In contrast, androgen-resistant tumors lack cathepsin L expression, with the p200 CUX1 variant being the dominant isoform. Knock-down of p200 in these cathepsin L-negative cells enhanced rather than suppressed invasiveness. This indicates that p110 promotes tumor progression, whereas p200, in the absence of cathepsin L, might exert tumor-suppressive actions [47]. It may be hypothesized that the delayed but also tumor-promoting phenotype seen in our p200 CUX1 mouse model is mediated by cathepsin L converting p200 into the active, tumor-promoting p110 variant.

In pancreatic cancer, several reports indicate that epidermal growth factor receptor (EGFR) signaling remains active and essential for tumor development despite downstream KRAS activation. Numerous downstream effectors of EGFR signaling bypassing activated KRAS and promoting tumor progression have been identified, including NFATc1 [48] and c-MYC [49]. Moreover, Ardito et al. showed that EGFR was required for KRAS-induced pancreatic tumorigenesis: Without EGFR activity, active RAS levels were not sufficient to induce robust MEK-ERK activity. The authors also showed that activation of EGFR was dependent on an EGFR ligand sheddase, ADAM17 [34], which is a member of the a disintegrin and metalloprotease (ADAM) family of proteases consisting of 21 members. ADAM17 is responsible for cleavage and release of a broad spectrum of substrates at the cell surface, including EGFR ligands, soluble TNFα, cytokines and adhesion molecules [50]. Thereby, ADAM17 is able to enhance activation of several oncogenic signaling pathways, including EGF–EGFR signaling.

Our data showed that p110 CUX1 significantly induced ADAM17 protein levels and activity both in vitro and in vivo. This indicated a strong link between p110 CUX1 and enhanced EGF signaling induced by ADAM17: CUX1 induces ADAM17 expression, which in turn proteolytically activates EGF family members such as amphiregulin (AREG) that induce EGFR signaling and its downstream cascade. EGFR-dependent pathways activate MEK-ERK and other downstream effectors, thereby driving tumor cell proliferation (Figure 8). Since ADAM17 upregulation was only seen on the protein level, additional post-transcriptional mechanisms might be involved downstream of CUX1. MEK-ERK activation itself has also been described to induce ADAM17 phosphorylation, thereby leading to stabilization of the protein. It is likely that upregulation of ADAM17 on the protein level, readily detected in both murine tissues and all cell lines, is mediated by this CUX1-induced MEK-ERK-ADAM17 feedback loop.

Several downstream kinases have been described to activate MEK-ERK signaling independently of EGFR or KRAS mutational status, among them the c-mos proto-oncogene product MOS, a serine/threonine kinase that directly phosphorylates MAPK-ERK kinase (MEK) [36]. We identified MOS as transcriptional target of p110 CUX in pancreatic cancer cells in vitro and in vivo. Supporting its important oncogenic role independent of upstream EGFR signaling, MOS has been described in lung cancer models as essential activator of both MEK-ERK and phosphoinositide 3-kinase (PI3K)-AKT pathways in an EGFR-independent manner, compensating for the loss of EGFR activity [51]. Our data indicated that CUX1-induced MOS acted despite the presence of activated Kras. These findings were in line with other reports in lung cancer that observed a mutually exclusive relationship between mutated KRAS and MOS overexpression driving cancer progression [52]. Interestingly, an early report by Higgy et al. has already described a direct interaction of CUX1 with the c-mos promoter region in germ cells. In this paper, binding assays using full-length p200 CUX1 resulted in c-mos suppression, contrary to our findings indicating transcriptional upregulation of MOS as a protumoral downstream effector of p110 CUX1 [53]. Further experiments are warranted to investigate a potential isoform-specific differential regulation of MOS by CUX1. In this context, it may be speculated that p110 CUX1 acts as activator and p200 as repressor of MOS.

## 5. Conclusions

Taken together, our data identified CUX1 as an important enhancer of KRAS-induced tumor development in pancreatic cancer, acting synergistically with oncogenic KRAS by inducing several upstream and downstream effectors such as ADAM17 and MOS. CUX1-induced signaling led to enhanced MEK/ERK activation, increased proliferation, accelerated PanIN formation and development of invasive pancreatic cancers. Based on our in vitro and in vivo data, the CUX1–EGF–MEK-ERK circuit might represent a promising target for therapeutic intervention to target KRAS-driven pancreatic cancer.

## Data Availability

All data generated or analyzed during this study are included in this published article and its supplementary information files.

## Supplementary Materials

The following are available online at https://www.mdpi.com/article/10.3390/cancers13102462/s1: Figure S1. CUX1 expression during KRAS-driven mPanIN development, Figure S2. p200 CUX1 has no impact on survival during KRAS-driven PDAC development, Figure S3. Generation and verification of Cux1p110 mice, Figure S4. p110 CUX1 potentiates KRAS-driven proliferation during PanIN progression, Figure S5. p110 CUX1 potentiates KRAS-driven proliferation during PanIN progression, Figure S6. Expression of p110 CUX1 in p200 CUX1-overexpressing Panc-1 cells, Figure S7. p110 CUX1 mRNA expression in pancreatic cell lines, Figure S8. p110 CUX1 enhances colony formation in mutant KRAS expressing pancreatic cell lines, Figure S9. Uncropped Western Blot images, Figure S10. Densitometry analysis of Western Blots.
