# Supplementary material for: CUX1 Enhances Pancreatic Cancer Formation by Synergizing with KRAS and Inducing MEK/ERK-Dependent Proliferation
Source: Cancers (Basel). 2021 May 18;13(10):2462. doi: 10.3390/cancers13102462 (PMC8158495; doi:10.3390/cancers13102462)
Supplement: Supplementary file 1 [file cancers-13-02462-s001.zip › cancers-1188213-supplementary.pdf]

Supplementary Materials: CUX1 Enhances Pancreatic Cancer Formation by Synergizing with KRAS and Inducing MEK/ERK-Dependent Proliferation

Heidi Griesmann, Sebastian Mühl, Jan Riedel, Katharina Theuerkorn, Bence Sipos, Irene Esposito, Gregory B. Vanden Heuvel and Patrick Michl

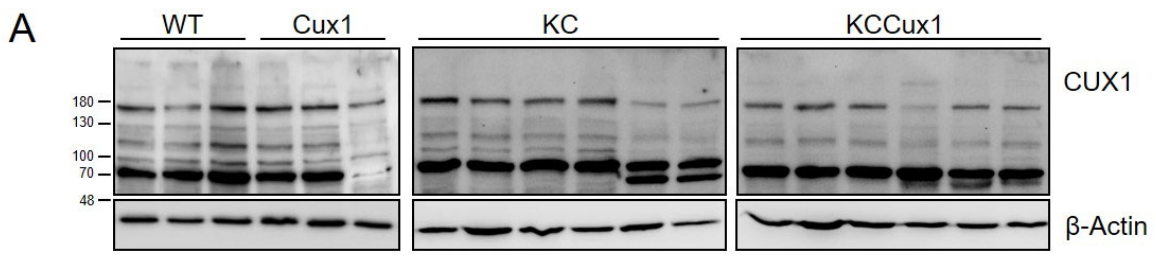

Figure S1. CUX1 expression during KRAS-driven mPanIN development. Western blot analysis of whole pancreatic protein lysates for CUX1 from 3-month-old WT, Cux1, KC and KCCux1 mice. Actin served as control. The uncropped Western Blot images can be found in Figure S9 and the densitometry analysis can be found in Figure S10.

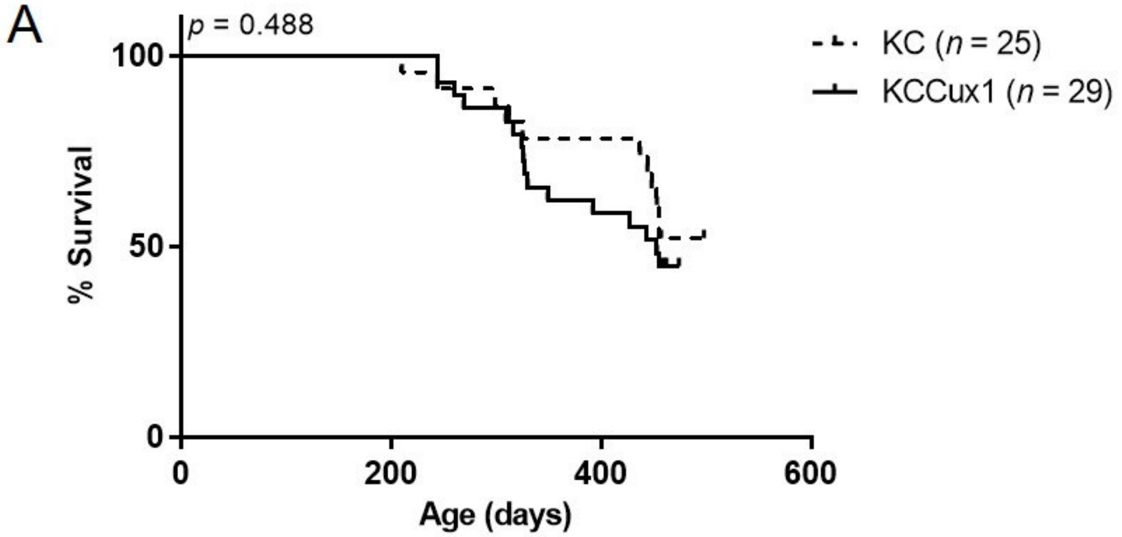

Figure S2. p200 CUX1 has no impact on survival during KRAS-driven PDAC development. Survival of KC (n = 25) and KCCux1 (n = 29) mice. Kaplan–Meier curves show a median survival for KC mice of 457 days and for KCCux1 mice of 453 days without significant difference by log-rank test.

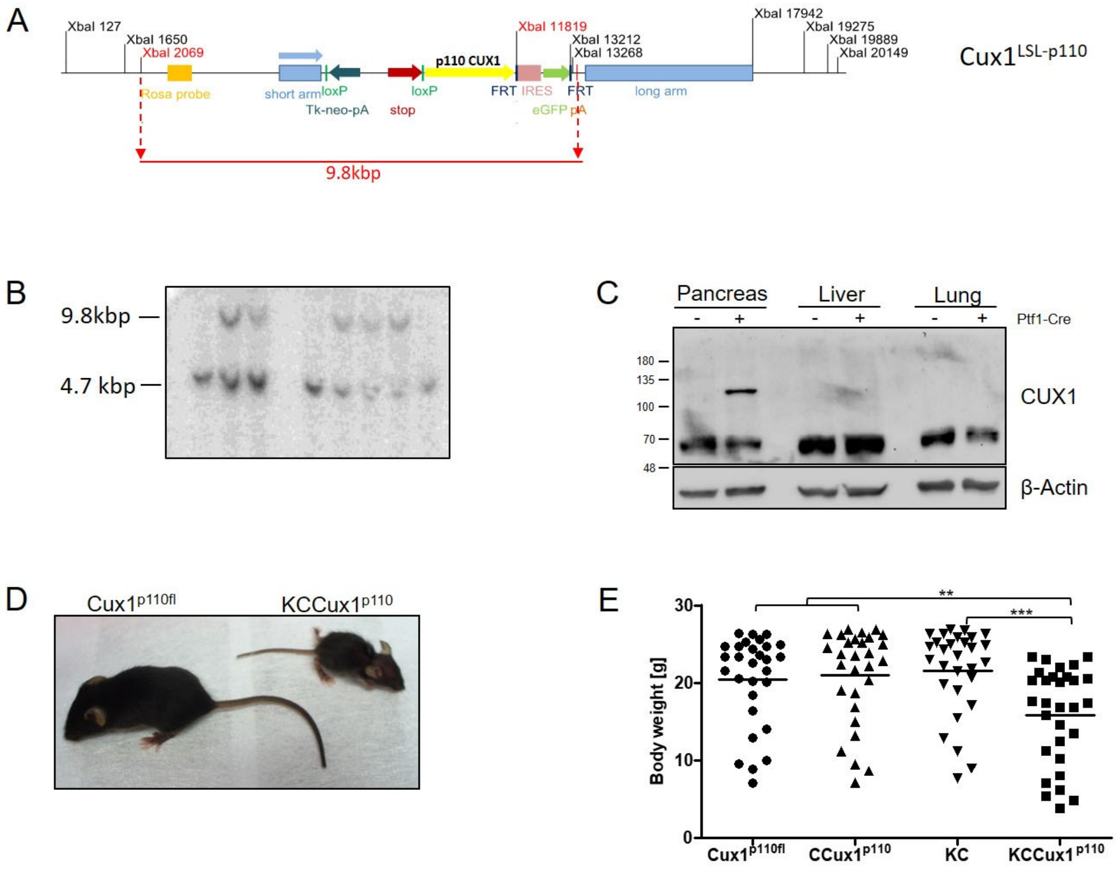

Figure S3. Generation and verification of Cux1p110 mice. (**A**) Targeting vector for the introduction of p110CUX1 into the Rosa26 locus to generate the CUX1LSL-p110/+ mouse line. P110 CUX1 is shown in yellow, the STOP cassette in red and the eGFP reporter in light green. The sites for homologous recombination are illustrated in blue. Restriction sites XbaI and specific probe are indicated in red. (**B**) Southern blot analysis of ES cell clones with the Rosa26 specific probe after XbaI digest. Positive clones show a signal at 9.8 kbp, which is indicative for the targeted allele, and an additional allele at 4.7 kbp signal, indicative for the WT allele. (**C**) Western blot analysis of whole lysates from pancreas, liver and lung shows a restricted expression of p110CUX1 in a Ptf1aCre/+; Cux1LSL-110/+ (CCux1p110) mouse (+) compared to a CUX1LSL-p110/+ mouse (−) without Ptf1a-Cre. (**D**) Delayed growth of KrasLSL-G12D/+; Ptf1aCre/+; Cux1LSL-p110/+ (KCCux1p110) mice compared to a wild-type sibling at the age of 4 weeks. (**E**) The body weights of both genders from KCCux1p110 (n = 9), KC (n = 6), CCux1p110 (n = 6) and Cux1p110fl (n = 6) mice measured at regular intervals after birth started at day 15 till day 84. The uncropped Western Blot images can be found in Figure S9 and the densitometry analysis can be found in Figure S10.

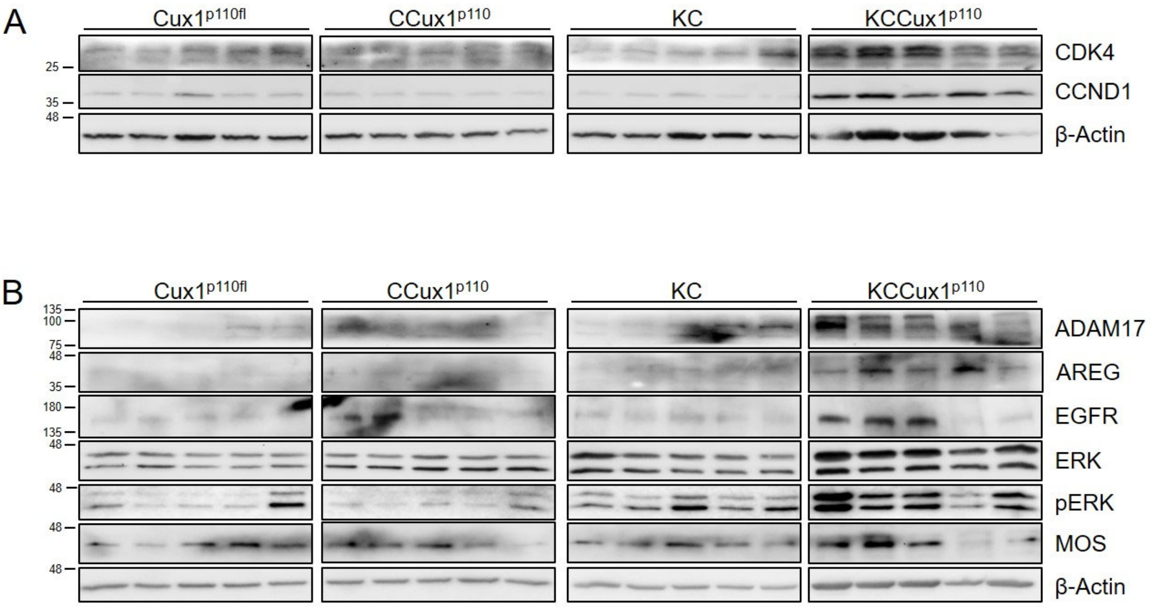

Figure S4. p110 CUX1 potentiates KRAS-driven proliferation during PanIN progression. **A–B** Western blot analysis of whole pancreas protein lysates from 3-month-old control Cux1p110, CCux1p110, KC and KCCux1p110 mice for proliferation markers CCND1 and CDK4 (**A**) as well as for the upstream signaling effector axis ADAM17-AREG-EGFR and their downstream effectors ERK, pERK and MOS (**B**)**.** Actin served as control. The uncropped Western Blot images can be found in Figure S9 and the densitometry analysis can be found in Figure S10.

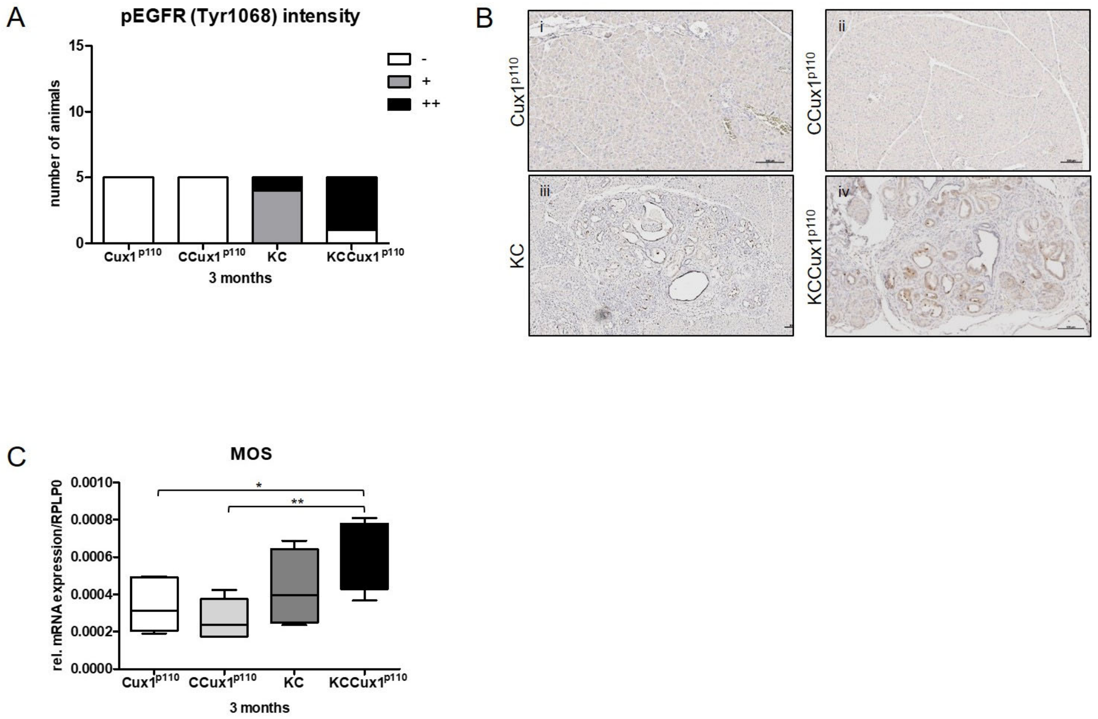

Figure S5. p110 CUX1 potentiates KRAS-driven proliferation during PanIN progression. (**A**) Quantification of pEGFR (Tyr1068) intensity in pancreatic tissue slides from 3-month-old control Cux1p110, CCux1p110, KC and KCCux1p110 mice (each, n = 5). Immunohistochemistry intensity score: + weak, ++ moderate, +++ strong. (**B**) Representative immunohistochemistry of pEGFR (Tyr1068) in pancreatic sections obtained from 3-month-old Cux1p110 (i), CCux1p110 (ii), KC (iii) and KCCux1p110 (iv) animals. Scale bar, 100 µm. (**C**) Quantitative RT-PCR analysis of MOS mRNA in 3-month-old control Cux1p110, CCux1p110, KC and KCCux1p110 mice (each, n = 5) normalized to the ribosomal protein RPLP0 as internal standard. *$p \leq 0.04$; **$p \leq 0.009$ by t-test.

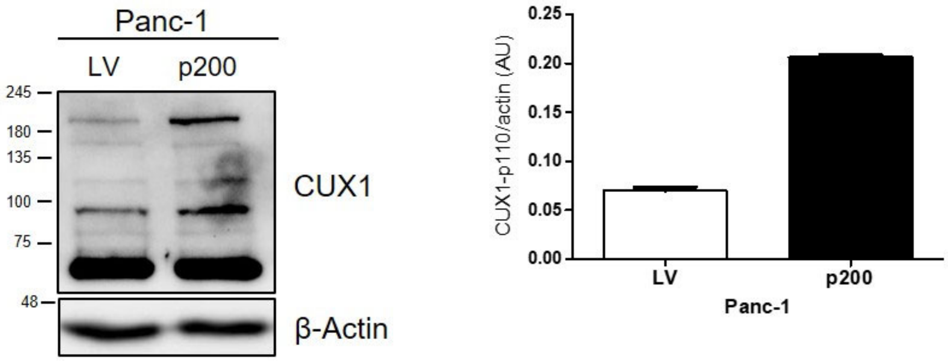

Figure S6. Expression of p110 CUX1 in p200 CUX1-overexpressing Panc-1 cells. Detection of the p110 CUX1 variant in whole protein cell lysates of Panc-1 cells stably transduced with p200 CUX1. Western blot shows clear p200 overexpression and an additional p110 CUX1 band in Panc-1-p200 CUX1 cells compared to Panc-1-LV (empty vector) cells. Actin served as control. Quantitative densitometry (right) illustrates the increased p110 CUX1 protein expression in Panc-1-p200 CUX1 cells compared to Panc-1-LV cells. Data represent three densitometries and were normalized to the intensity of actin bands. Arbitrary units (AU) are expressed as mean ± SD. ***$p < 0.0001$. Symbols: LV = empty vector; p200 = p200 CUX1. The uncropped Western Blot images can be found in Figure S9 and the densitometry analysis can be found in Figure S10.

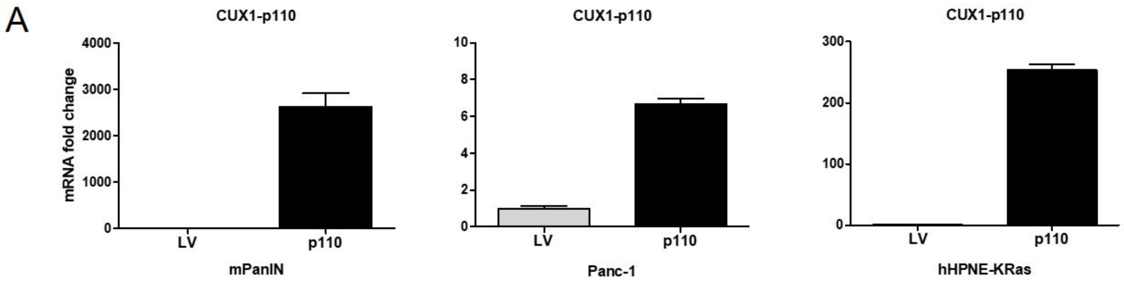

Figure S7. p110 CUX1 mRNA expression in pancreatic cell lines. Representative analysis of p110 CUX1 in empty vector (LV) and p110 CUX1 overexpressing mPanIN, Panc-1 and hHPNE-KRAS cells. Quantitative RT-PCR was normalized to the ribosomal protein RPLP0 as internal standard.

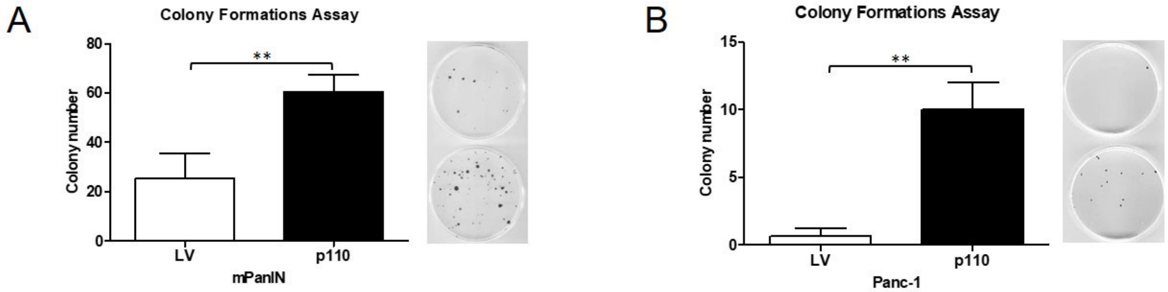

Figure S8. p110 CUX1 enhances colony formation in mutant KRAS expressing pancreatic cell lines. **A**–**B** p110 CUX1 over-expression increased colony formation in mPanIN (**A**) and Panc-1 (**B**) cells compared to their empty vector-transduced control (LV) cells. A representative crystal violet staining is shown on the right. Graphs represent the mean ± SD of three independent experiments. **$p \leq 0.0015$; by Student's t-test.

Figure 4B

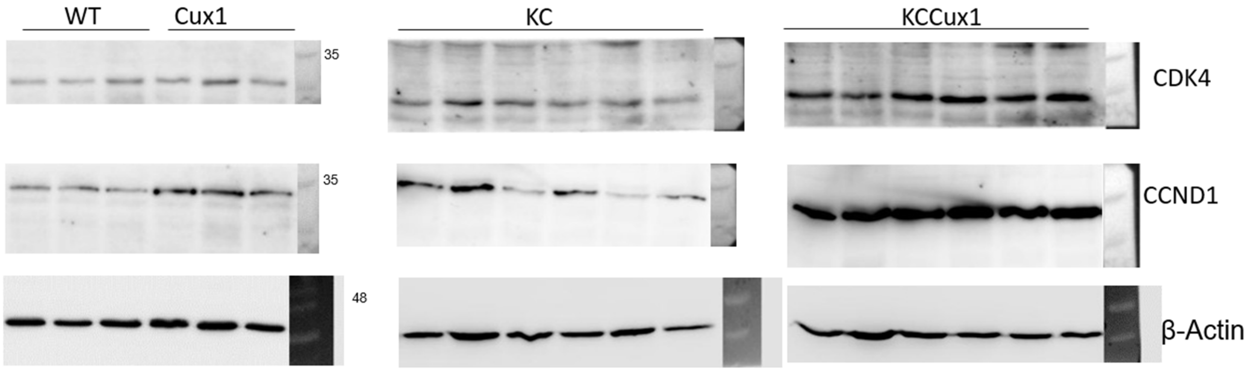

Figure 4C

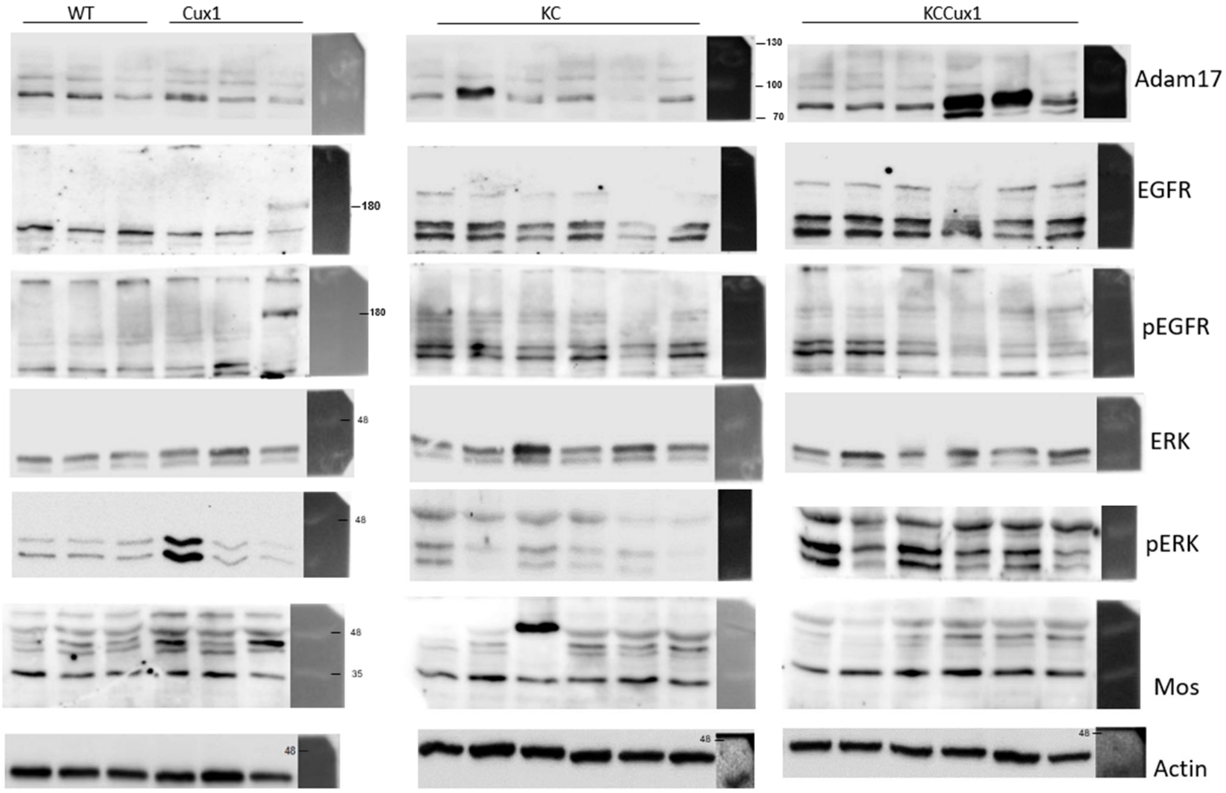

Figure 5A

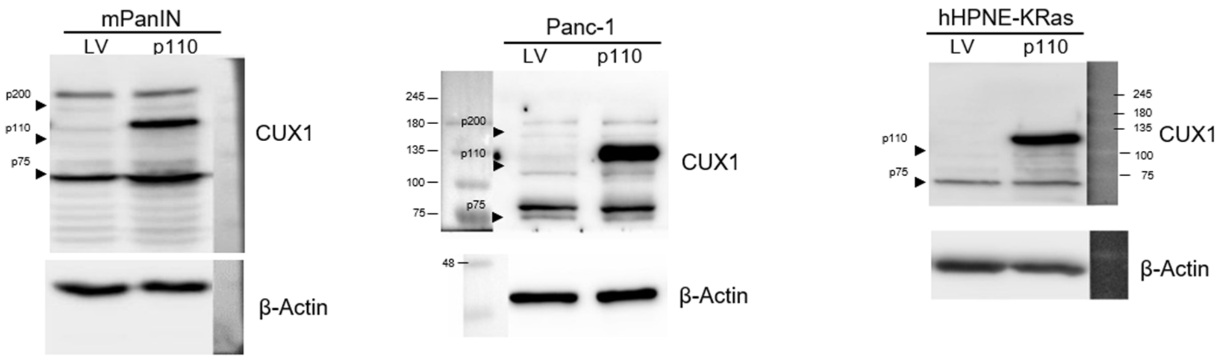

Figure 6A

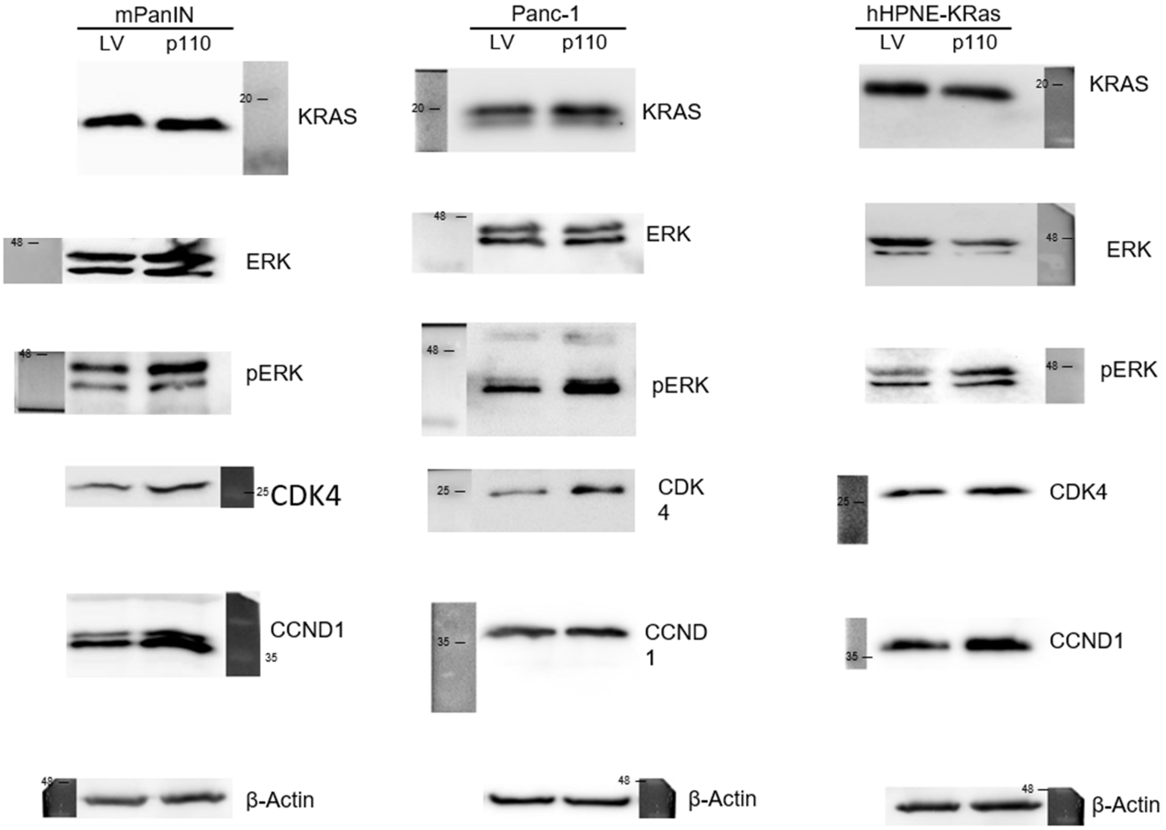

Figure 6B

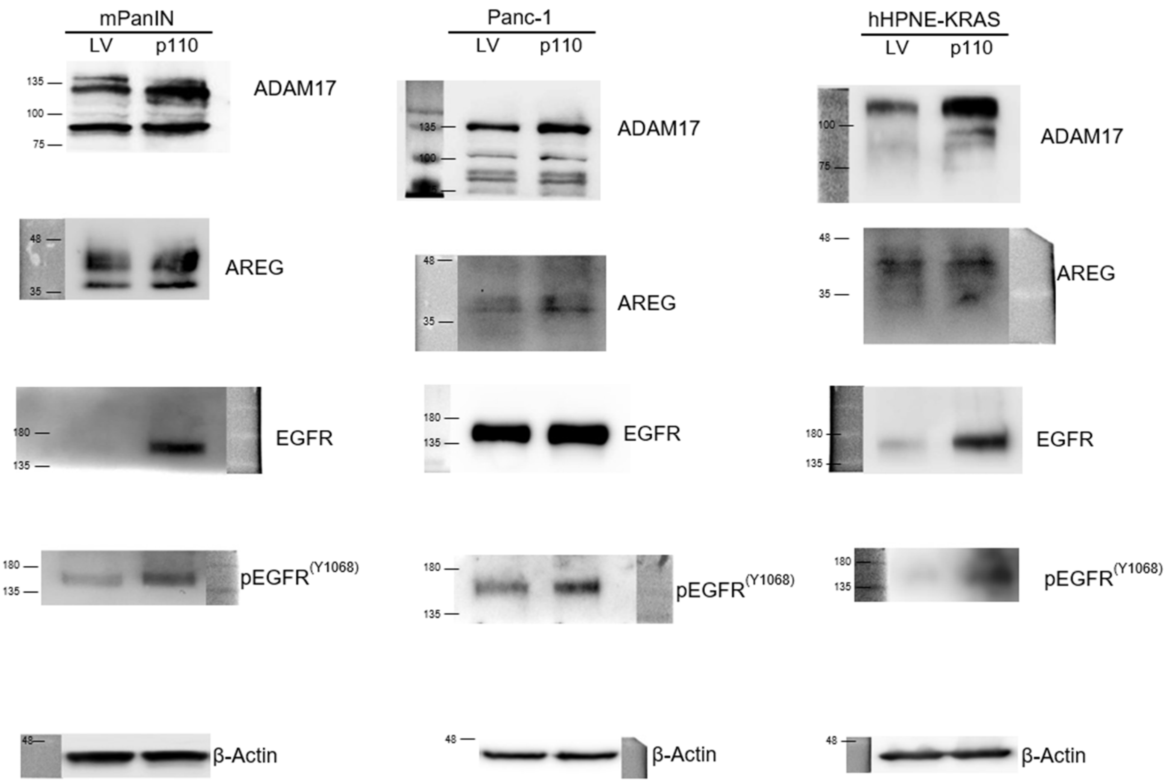

Figure 7A

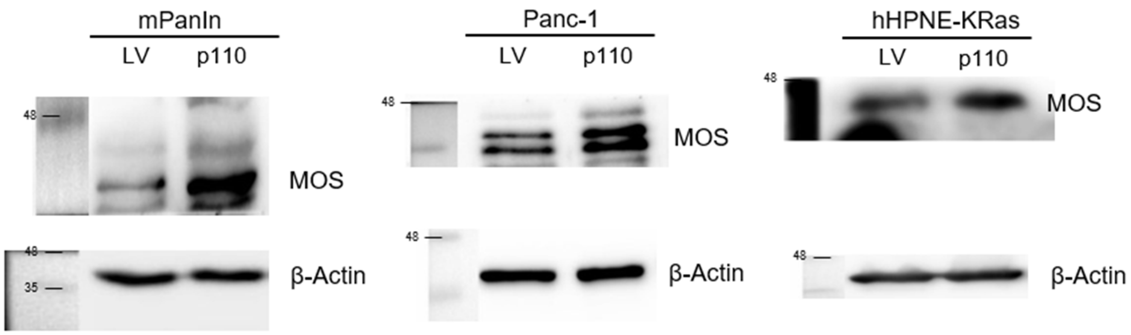

Figure S1

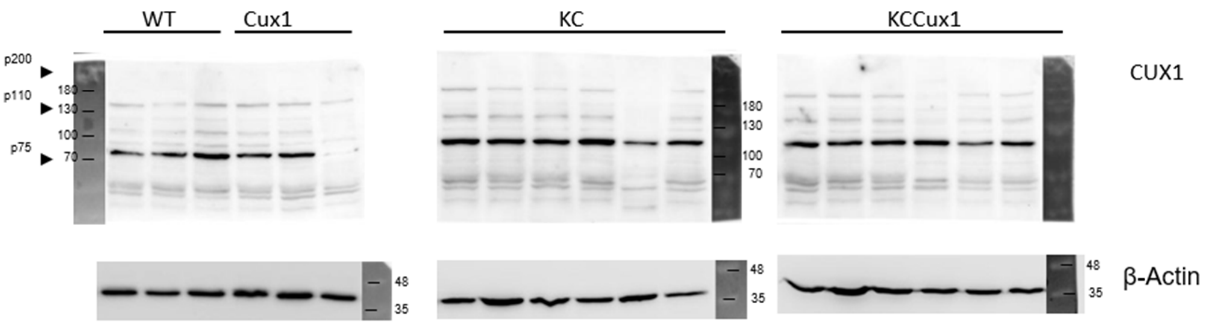

Figure S3

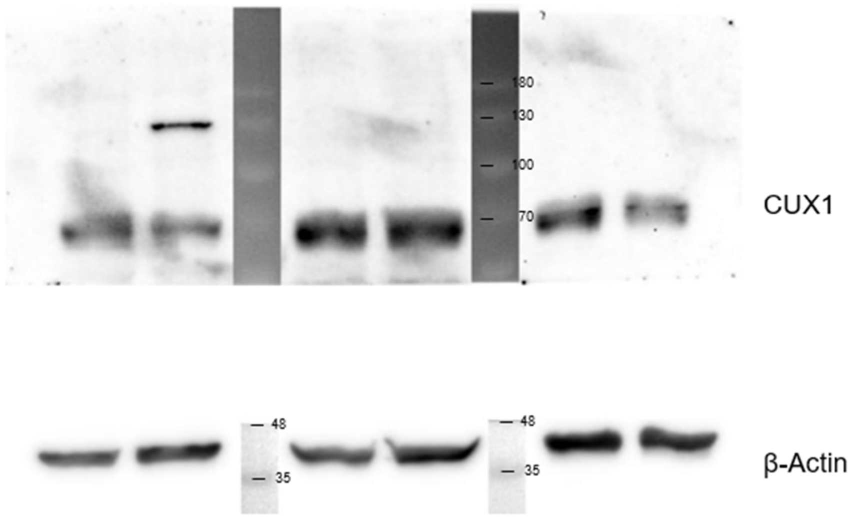

Figure S4A

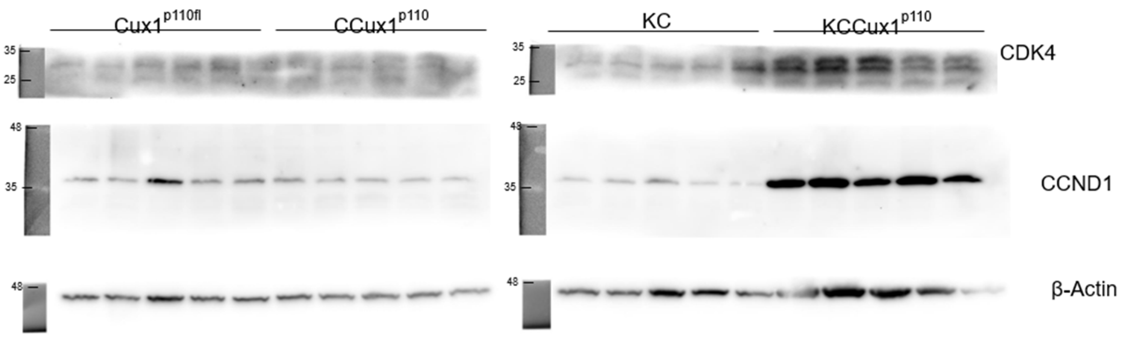

Figure S4B

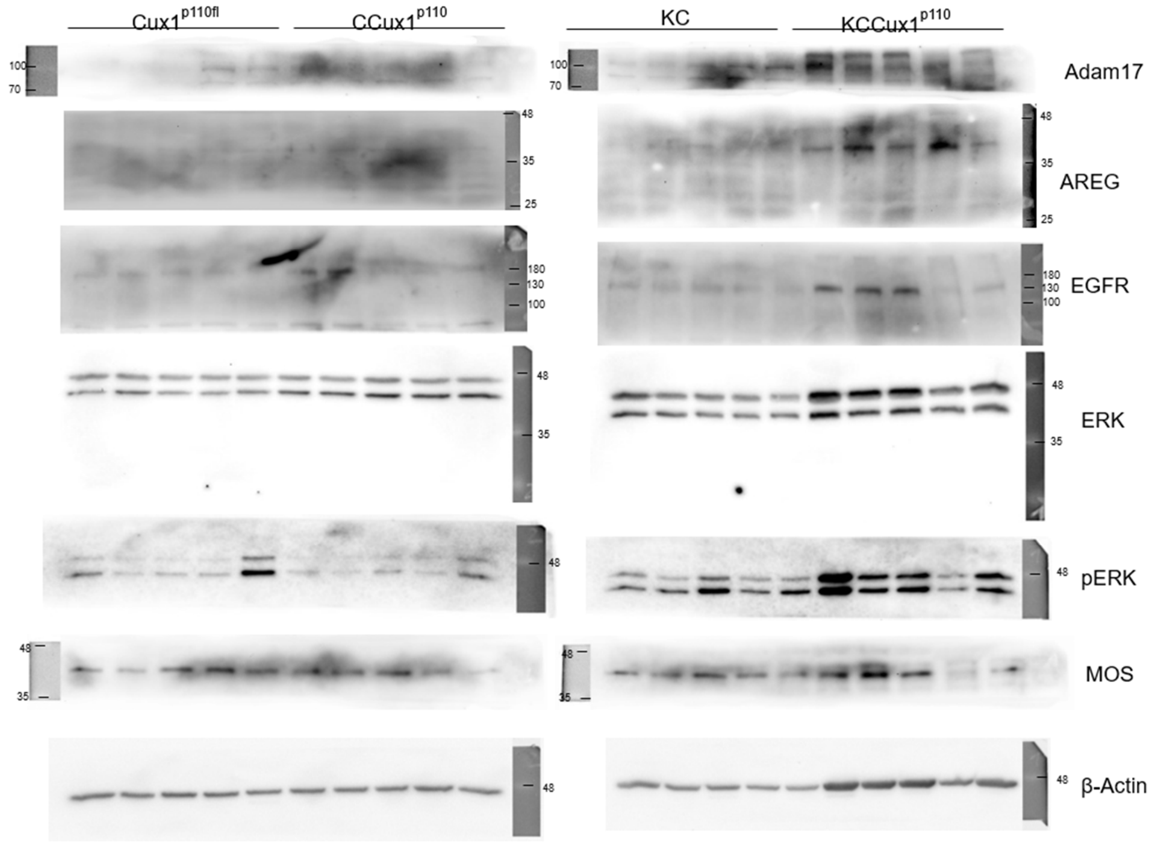

Figure S6

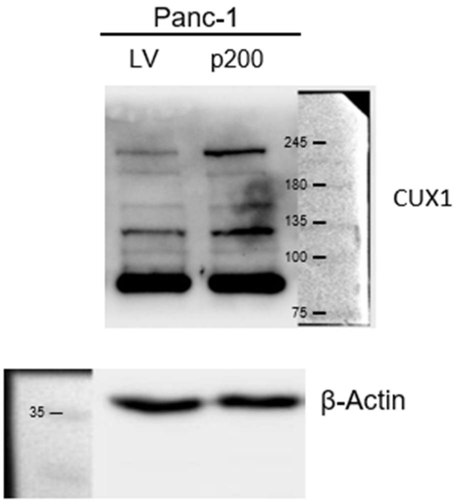

Figure S9. Uncropped Western Blot images.

Figure 4B

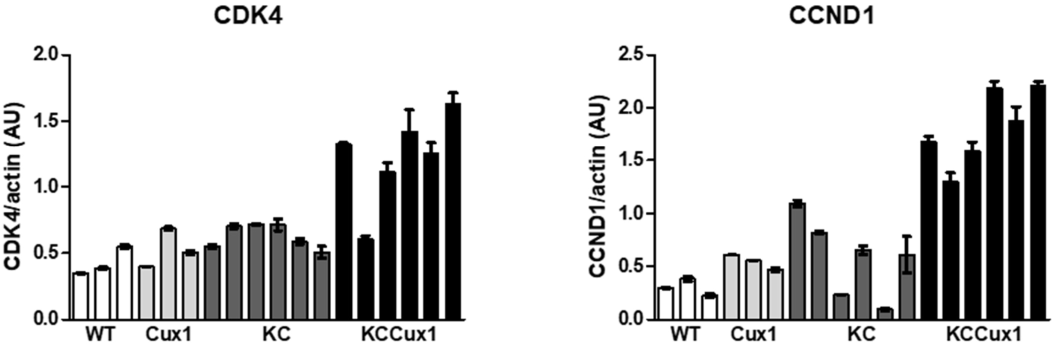

Figure 4C

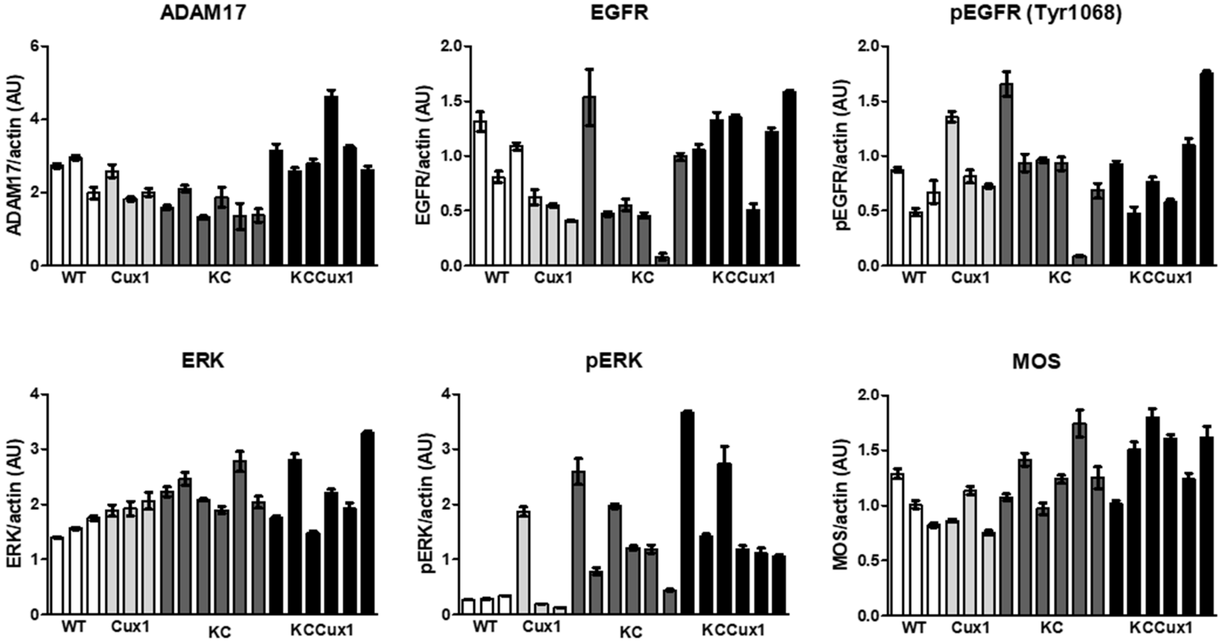

Figure 5A

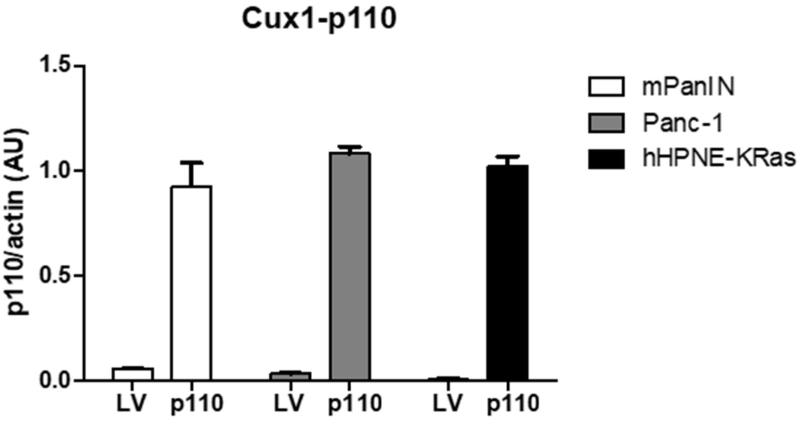

Figure 6A

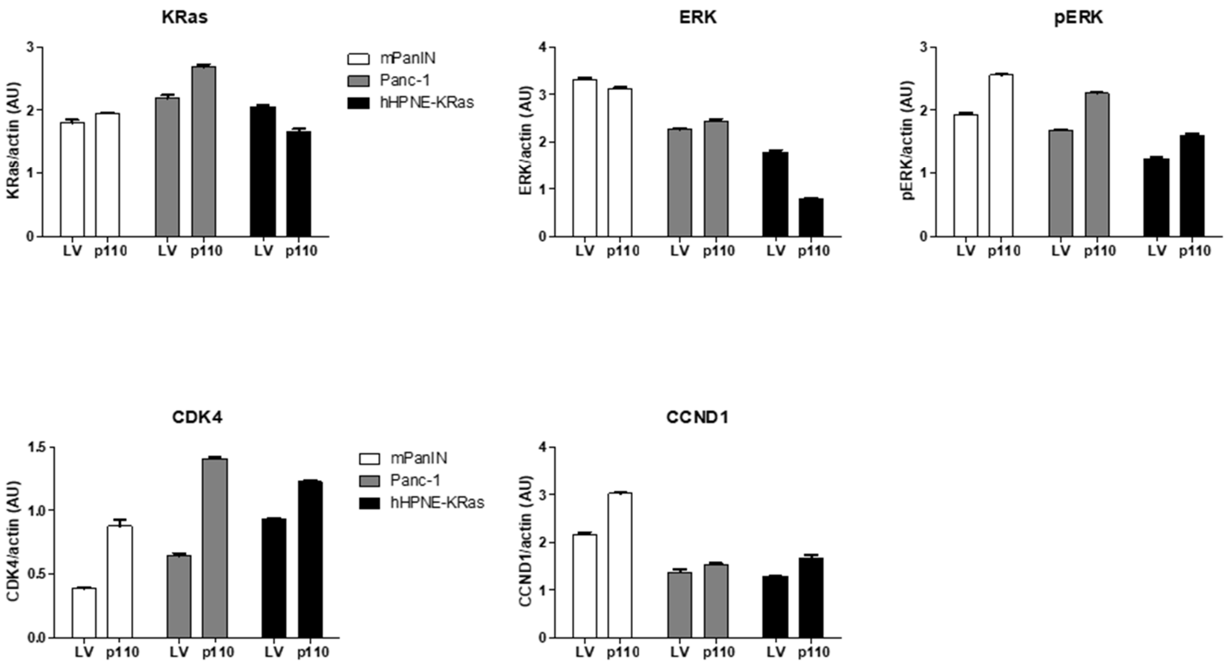

Figure 7A

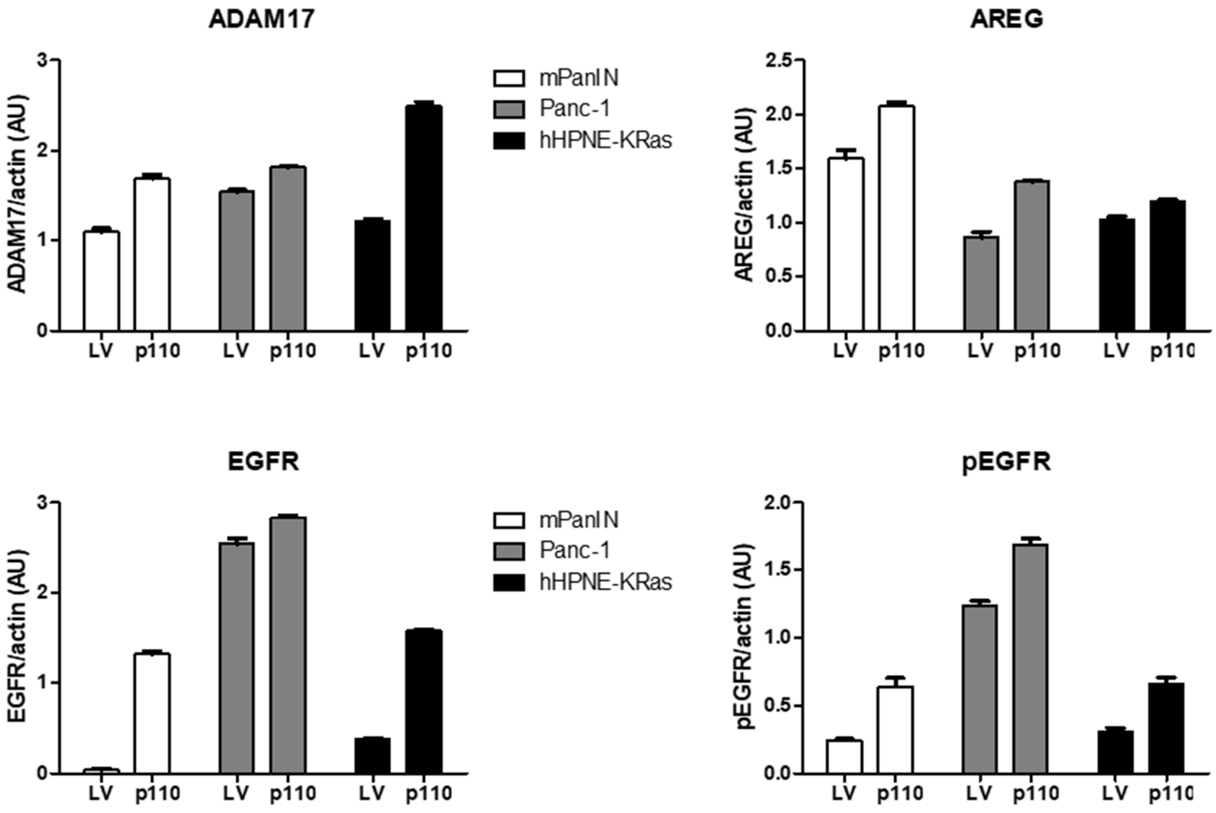

Figure 8A

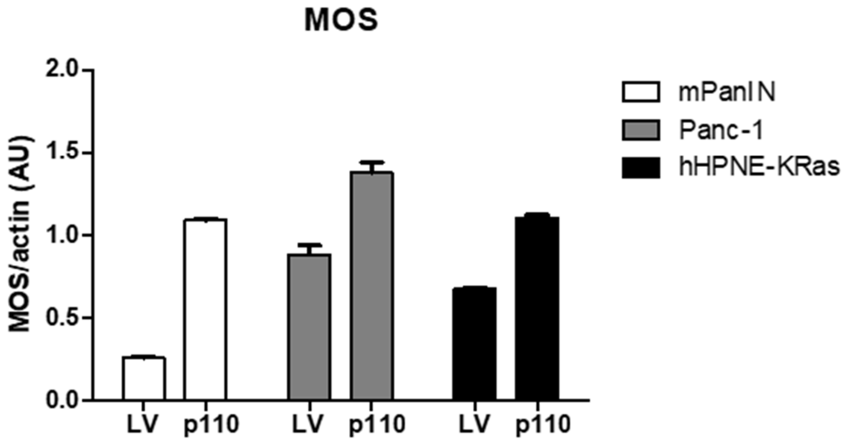

Figure S1A

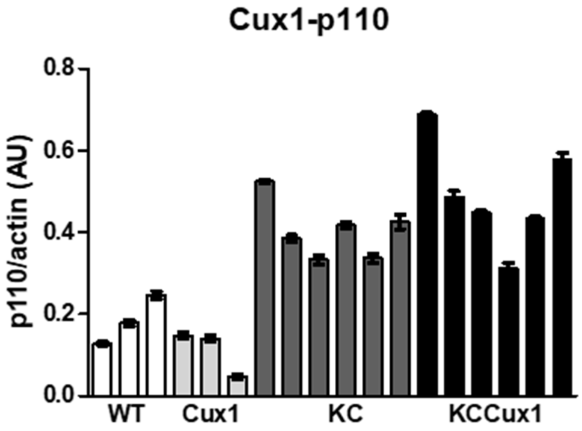

Figure S3B

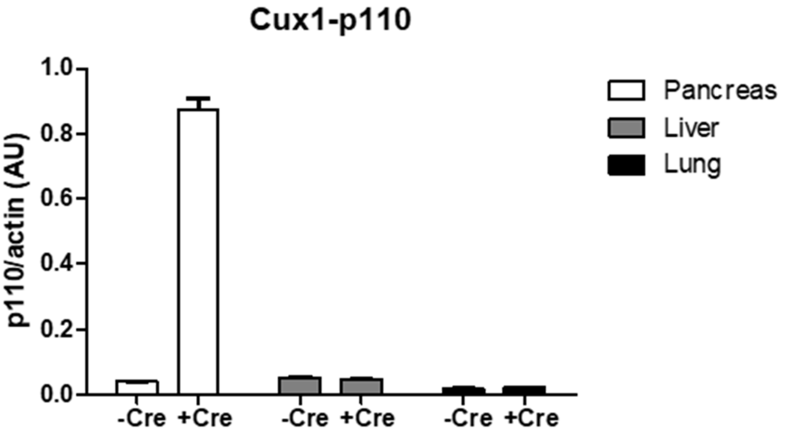

Figure S4A

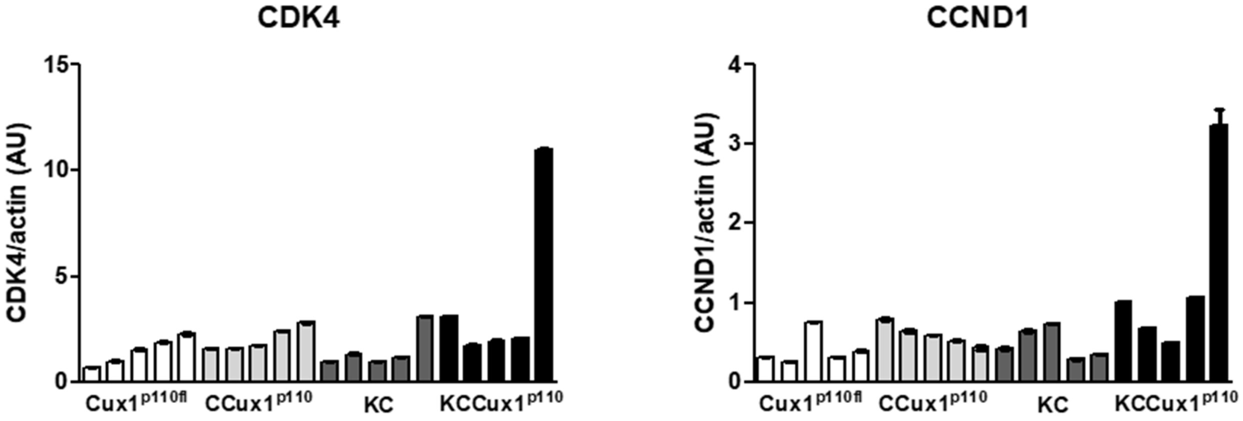

Figure S4B

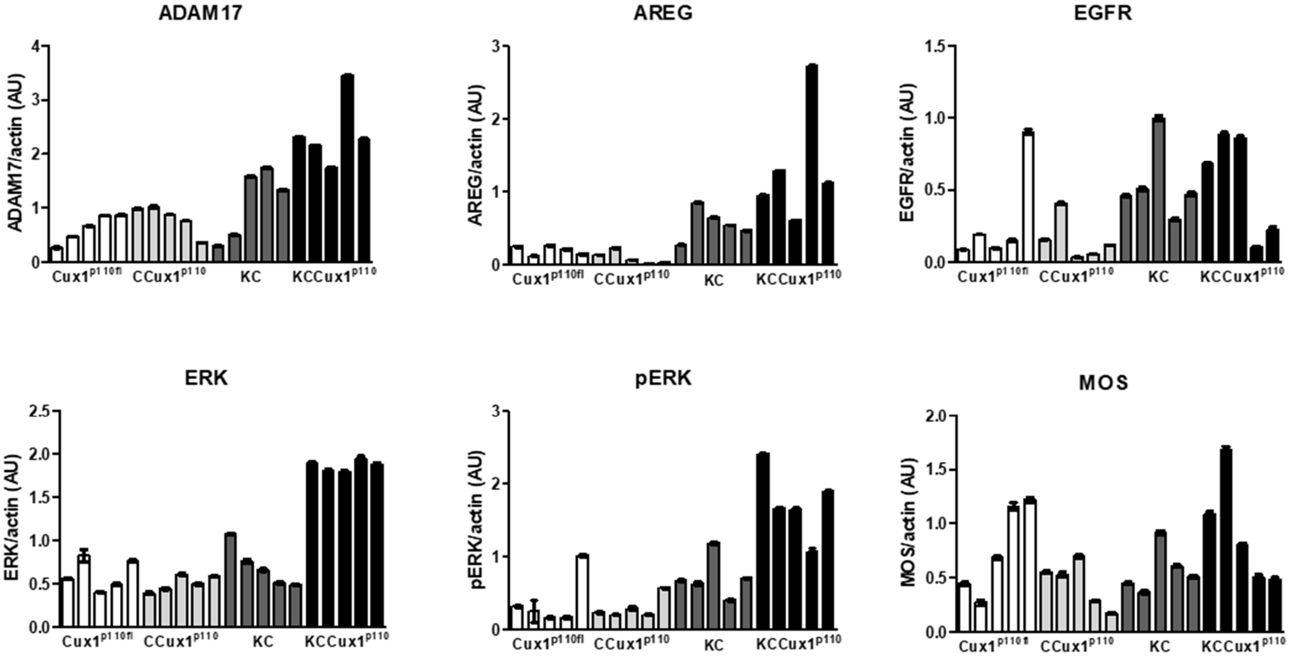

Figure S10. Densitometry analysis of Western Blots.
